# Establishing conserved biosynthetic gene clusters of the phylum Myxococcota

**DOI:** 10.1128/aem.02151-25

**Published:** 2025-12-11

**Authors:** Shailaja Khanal Pokharel, Nawal Shehata, Andrew Ahearne, Thomas Knehans, Constance B. Bailey, Paul D. Boudreau, D. Cole Stevens

**Affiliations:** 1 Department of Biomolecular Sciences, School of Pharmacy, University of Mississippi 8083, Oxford, Mississippi, USA; 2 School of Chemistry, University of Sydney, Camperdown, New South Wales, Australia; Danmarks Tekniske Universitet The Novo Nordisk Foundation Center for Biosustainability, Kgs. Lyngby, Denmark

**Keywords:** myxobacteria, biosynthetic gene clusters, specialized metabolism, genome mining

## Abstract

A surge in sequenced myxobacteria catalyzed by advancements in long-read genome and metagenome sequencing has provided sufficient data to scrutinize the conserved biosynthetic gene clusters (BGCs) within the phylum Myxococcota. Provided the utility of myxobacteria in environmental nutrient cycles and discovery of novel therapeutic leads, we sought to determine any conserved specialized metabolism in the phylum. Using a pan-genome approach to analyze 11 genera and 195 sequenced genomes, including 10 newly reported myxobacterial isolates, we observed five conserved BGCs. All five clusters encode for characterized metabolites with established ecological roles for four of the metabolites, and none of the metabolites are known toxins. Validation of our approach was done by analyzing Myxococcota genera without sufficient sequenced representatives for pan-genome analysis to observe the presence/absence of these five clusters. This approach enabled observation of genus-level conservation of BGCs with varying degrees of confidence due to the diversity of sequenced species within each genus. The indigoidine BGC typically found in *Streptomyces* spp. was notably conserved in *Melittangium*; heterologous expression of the core biosynthetic gene *bspA* in *Escherichia coli* and subsequent detection of indigoidine confirmed the identity of the indigoidine cluster. Conserved BGCs in myxobacteria reveal maintenance of biosynthetic pathways and cognate metabolites with ecological roles as chemical signals and stress response; these observations suggest competitive specialization of secondary metabolism and toxin production in myxobacteria.

**IMPORTANCE:**

Critical contributions to soil nutrient cycles by predatory bacteria, including the Myxococcota, and utility as a resource for the discovery of novel enzymology and metabolism motive continued isolation and characterization of myxobacteria from the environment. Each of these motivating factors involves specialized metabolites produced by myxobacteria and the biosynthetic gene clusters (BGCs) responsible for their assembly. Primarily associated with the predatory lifestyles of myxobacteria, myxobacterial specialized metabolites have been pursued as therapeutic leads for novel antibacterials, antifungals, anthelmintics, and cancer therapies. Despite these efforts and the observation that nearly all genera within the Myxococcota have an extraordinary number of BGCs, there is no consensus view of the conserved BGCs in the phylum. Our study revealed the core BGCs consistently present throughout the phylum. By reporting these core specialized metabolites and their ecological roles, we hope to streamline the discovery and investigation of specialized metabolism in myxobacteria.

## INTRODUCTION

Myxococcota possess numerous features that motivate continued discovery of environmental isolates, such as cosmopolitan, predatory lifestyles that contribute to soil nutrient cycles and extraordinary capacities to produce specialized metabolites with potential applications as therapeutic leads (1–5). A recent expansion of the phylum brought about by the isolation and characterization of novel species and genera has generated a wealth of genomic data to be scrutinized. Bioinformatic analyses have previously revealed that all genera within the phylum, excluding *Vulgatibacter* (6), accommodate numerous biosynthetic gene clusters (BGCs) responsible for the production of specialized metabolites (7, 8), and metabolomic analysis has demonstrated a correlation between metabolic profiles and taxonomic distance of myxobacteria (9). Common BGCs repeatedly observed in myxobacterial genomes have been noted, such as the geosmin BGC (10). However, the distribution and conservation of specific BGCs from myxobacteria have not been reported. Establishing a conserved set of BGCs across the Myxococcota will improve dereplication of common clusters for future genome mining and inform efforts to determine the ecological role of specialized metabolites from myxobacteria.

Inspired by a pan-genome analysis of *Corallococcus* spp. that found the core pan-genome excluded the majority of BGCs observed in the genus (11), we sought to determine the distribution and conservation of BGCs from sequenced Myxococcota. Although numerous database-driven pipelines for BGC analysis exist to annotate biosynthetic genes and determine pathway novelty (12, 13), we opted for an initial pan-genome analysis to identify conserved genes that were subsequently mapped back to BGCs annotated by antiSMASH (14, 15). Using an approach that is initially BGC-agnostic, we hoped to minimize challenges associated with ambiguous similarity scores of BGCs across analysis pipelines, influence of intrinsic homologies of core biosynthetic features, and occurrences of multiple discrete clusters in individual BGCs annotated as “hybrids”. We analyzed a total of 195 myxobacterial genomes from 11 genera and found five BGCs to be generally conserved in myxobacteria.

## RESULTS

### Genome sequencing and phylogenetic analysis of 10 isolated myxobacteria

In an effort to increase the number of analyzed myxobacterial genomes, we sought to include genome data from 10 newly isolated myxobacteria in our pan-genome analysis. Myxobacterial isolates were obtained from rhizospheric soil samples using prey-baiting methodology as previously described (16, 17). A total of 10 environmental isolates were sequenced to broaden our pan-genome analysis (Table 1). Taxonomic assignments for sequenced isolates included three *Archangium*, one *Cystobacter*, two *Melittangium*, two *Myxococcus*, and two *Nannocystis*. Comparative genome analysis of newly obtained isolates versus type strain myxobacteria provided average nucleotide identity (ANI) and digital DNA–DNA hybridization values (dDDH) supported several isolates being novel species (Table 1). Adhering to established thresholds for determining novel species (18, 19), isolates PVMSAZ, D1P2, TKBC04, and BB12-2 are candidate novel species. However, TKBC04 and D1P2 are both the same species and share 99.9% ANI. Both isolates also share >97% ANI with *Melittangium primigenium*. We previously reported the misassignment of *Me. primigenium* as *Archangium primigenium* ATCC 29037 and feel compelled to note the candidate status of the novel species represented by strains D1P2 and TKBC04 until further effort is done to validly describe and resolve the type strain for these *Melittangium* (17). The low-quality assembly of BB12-2 (>100 contigs) decreases our confidence in it being a novel *Myxococcus* species. Of the three *Archangium* isolates, PVMSAZ, a candidate novel species, is most related to *Archangium gephyra* (90.6% ANI), NCHinoki1 is a subspecies of *Archangium lansingense*, and SCPoplar1 is a subspecies of *Ar. gephyra*. Isolates NCWS and Hickory4 are subspecies of *Cystobacter fuscus* and *Myxococcus fulvus,* respectively. Isolates MIELM and UBH4 are both subspecies of *Nannocystis pusilla*.

**TABLE 1:** Genome assembly and phylogenetic data for sequenced isolates with proposed novel species bolded

Isolate	Size (bp)	CDS	GC%	N50	L50	Contigs	dDDH (d4)	ANI
*Archangium* sp. NCHinoki1	13,608,435	11,298	68.2	13,506,606	1	2	35.5%; *Archangium gephyra*	98.7%; *Archangium lansingense*
*Archangium* sp. PVMSAZ	**10,952,989**	**9,658**	**69.6**	**10,843,764**	**1**	**2**	**40.6%; *Ar. gephyra***	**90.6%; *Ar. gephyra***
*Archangium* sp. SCPoplar1	13,446,571	10,895	68.7	13,291,844	1	2	45.6%; *Ar. gephyra*	95.8%; *Ar. gephyra*
*Cystobacter* sp. NCWS	12,322,754	14,750	68.6	12,003,056	1	3	60.8%; *Cystobacter fuscus*	95.2%; *Cy. fuscus*
*Melittangium* sp. D1P2^*a*^	**9,298,785**	**7,782**	**70.8**	**n/a^*b*^**	**1**	**1**	**29.5%; *Melittangium boletus***	**85.7%; *Me. boletus***
*Melittangium* sp. TKBC04^*a*^	**9,298,954**	**7,763**	**70.8**	**n/a**	**1**	**1**	**29.5%; *Me. boletus***	**85.7%; *Me. boletus***
*Myxococcus* sp. BB12-2	**10,892,721**	**9,003**	**69**	**130,546**	**23**	**148**	**30.2%; *Myxococcus llanfair...^c^***	**85.2%; *My. llanfair...***
*Myxococcus* sp. Hickory4	10,160,340	8,489	70.1	108,358	30	182	59.7%; *Myxococcus fulvus*	95.1%; *My. fulvus*
*Nannocystis* sp. MIELM	12,731,429	14,925	71.4	12,694,743	1	3	56.6%; *Nannocystis pusilla*	94.9%; *Na. pusilla*
*Nannocystis* sp. UBH4	12,213,092	11,265	71.7	n/a	1	1	55.4%; *Na. pusilla*	94.7%; *Na. pusilla*

^*a*^ Isolates *Me*. D1P2 and *Me*. TKBC04 are the same species and share 99.9% ANI...

^*b*^ n/a, not applicable.

^*c*^ *Myxococcus llanfairpwllgwyngyllgogerychwyrndrobwllllantysiliogogogochensis* abbreviated to *Myxococcus llanfair*...

### Pan-genome analysis of Myxococcota

**Fig 1:**
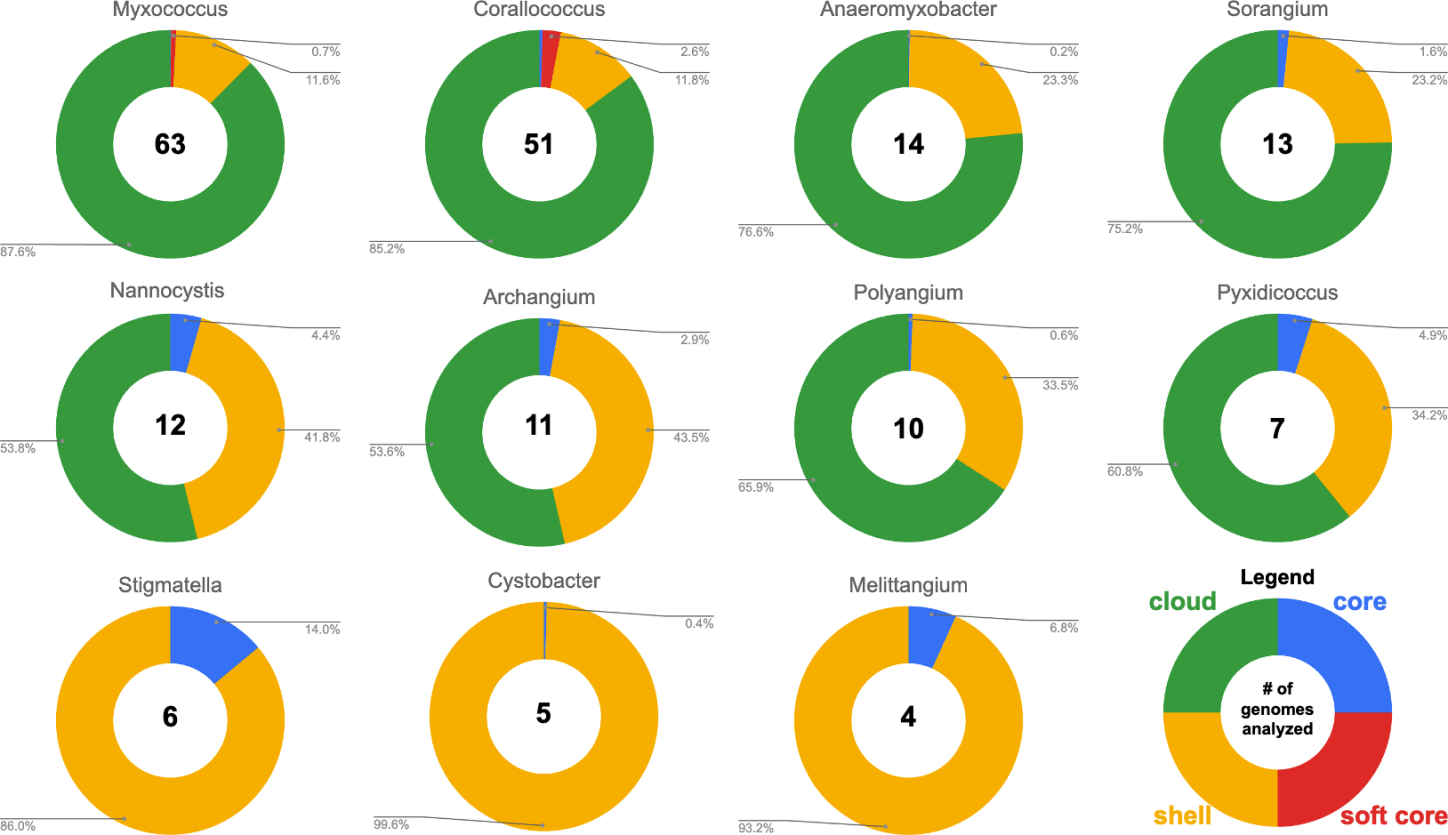
Summary of pan-genome analysis by genera. A complete list of myxobacteria included in our analysis can be found in Table S1.

Aiming to generate a list of conserved genes per genus, we analyzed the pan-genome of all sequenced genomes from each genus. The resulting list of genes conserved per genus served as a starting point to subsequently cross-reference with genes found to be present in BGCs to ultimately identify conserved BGCs. Pan-genomes for each Myxococcota genus were determined from a total of 195 genome assemblies with available genomes per genus ranging from 63 *Myxococcus* genomes to four *Melittangium* genomes (Fig. 1). Genera excluded from our analysis due to an insufficient number of sequenced representatives include *Aggregicoccus, Chondromyces*, *Citreicoccus*, *Enhygromyxa*, *Haliangium*, *Hyalangium*, *Kofleria*, *Labilithrix*, *Plesiocystis*, *Pseudenhygromyxa*, *Sandaracinus*, *Simulacricoccus*, *Vitiosangium*, and *Vulgatibacter*. Pan-genomes for members of the following genera were determined: *Myxococcus*, *Corallococcus*, *Anaeromyxobacter*, *Sorangium*, *Archangium*, *Nannocystis*, *Polyangium*, *Pyxidicoccus*, *Cystobacter*, *Stigmatella*, and *Melittangium* (Fig. 1). Resulting pan-genome data included core genes present in ≥99% of analyzed genomes, soft core genes present in 95%–98% of analyzed genomes, shell genes present in 15%–94% of analyzed genomes, and cloud genes present in <15% of analyzed genomes. Our analysis suggests all analyzed genera have open genomes with the majority of genes found to be in the accessory pan-genomes (shell and cloud). Clades with higher percentages of genes in core pan-genomes (*Melittangium* 6.8% and *Stigmatella* 14%) have fewer sequenced genomes available, and ANI values for members indicate these genera include genome data from highly related subspecies and fewer distinct species. For example, of the four *Melittangium* analyzed, only two distinct species with ANI and dDDH values below established thresholds are included in our analysis. The limited diversity of species in the *Cystobacter*, *Melittangium*, and *Stigmatella* clades likely impacts our assessment of conserved BGCs at the genus level. Despite this limitation, we retained and included all three genera with the caveat that the following BGCs conserved at the genus level for the *Cystobacter*, *Melittangium*, and *Stigmatella* may be overstated.

### Identification of five conserved BGCs in myxobacteria

**Fig 2:**
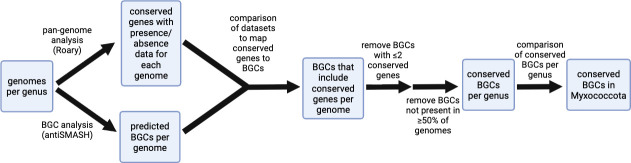
Methodological flow chart for identification of conserved BGCs in Myxococcota.

Following the flowchart in Fig. 2, we sought to identify BGCs conserved throughout Myxococcota. To do this, we utilized the list of conserved genes per genus from our pan-genome analysis to determine the extent of conserved genes present in BGCs identified by antiSMASH analysis. This step afforded a list with presence/absence data for core, soft core, and shell genes included in BGCs from each strain per genus. In an attempt to account for the differences in total number of strains analyzed per genus, we reduced the number of shell genes advanced by removing shell genes present in <50% of the analyzed genomes. This threshold was necessary to avoid consideration of genes present in a single strain from a genus with fewer than five sequenced genomes as conserved. This also enabled a rigorous, succinct cutoff for conserved genes present in BGCs to be genes conserved in ≥50% of strains within a genus. However, this threshold did not account for conserved genes that are present in BGCs of some strains but not others. To address this, we removed BGCs with fewer than two conserved genes from our analysis. The requirement that BGCs must include at least two conserved genes to be considered conserved in our analysis was selected after determining that an increase to remove BGCs with fewer than three conserved genes would only remove an additional three BGCs from our data set. We considered the remaining BGCs in our data set to be conserved in their respective genus. Finally, conserved BGCs from each genus were aggregated and used to assess the distribution of common BGCs within the phylum. AntiSMASH similarity scores and comparison with representative BGCs in the MiBIG database afforded assignments for characterized BGCs and associated metabolites (20, 21).

**Fig 3:**
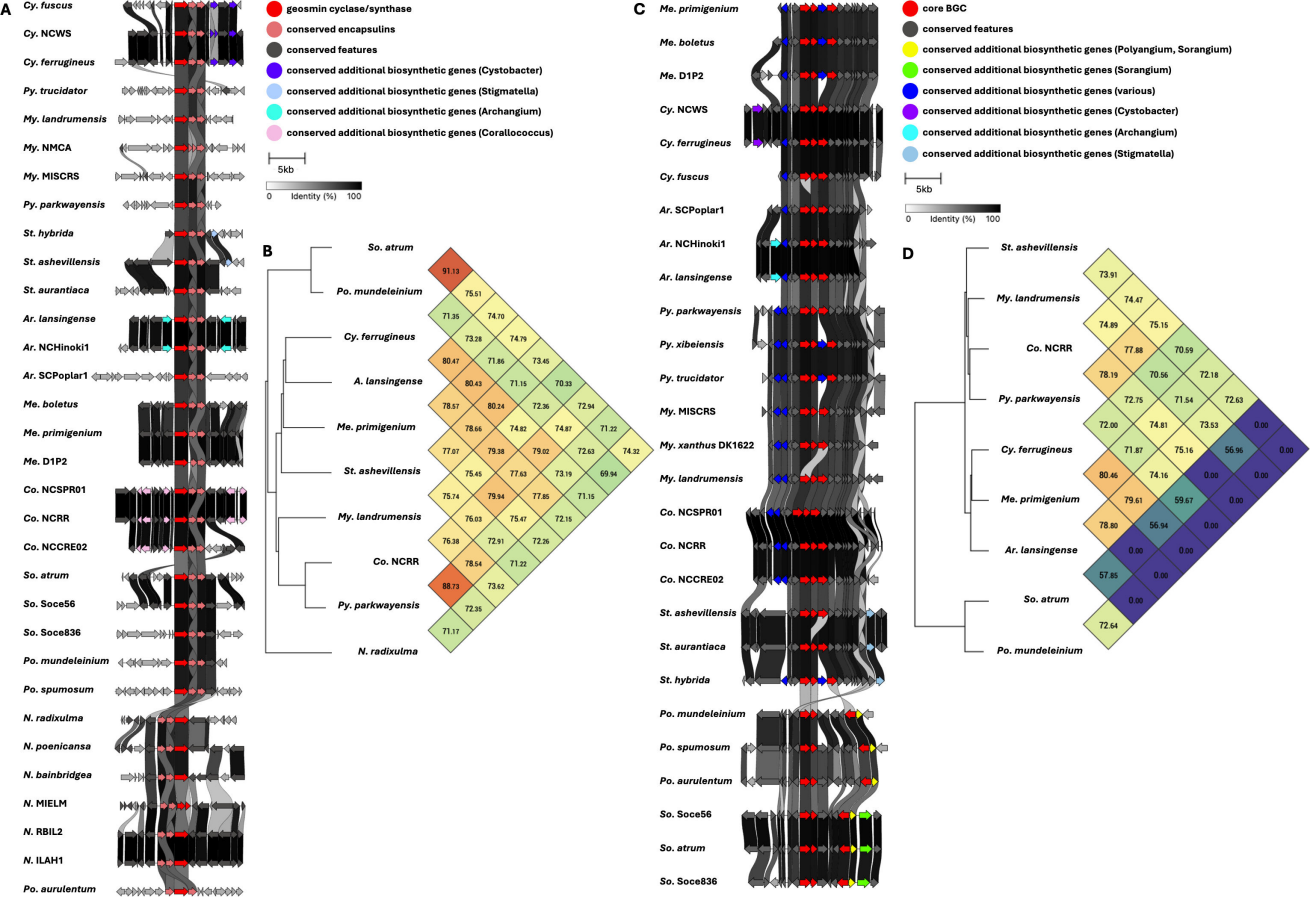
Conservation and genetic organization of the geosmin BGC in Myxococcota (**A**) and ANI values for geosmin BGCs across representatives of host genera (**B**). Conservation and genetic organization of the carotenoid BGC in Myxococcota (**C**) and ANI values for carotenoid BGCs across representatives of host genera (**D**). AntiSMASH analysis provided .gbk files utilized to generate images in clinker and ANI values using OrthoANI (32, 36).

**Fig 4:**
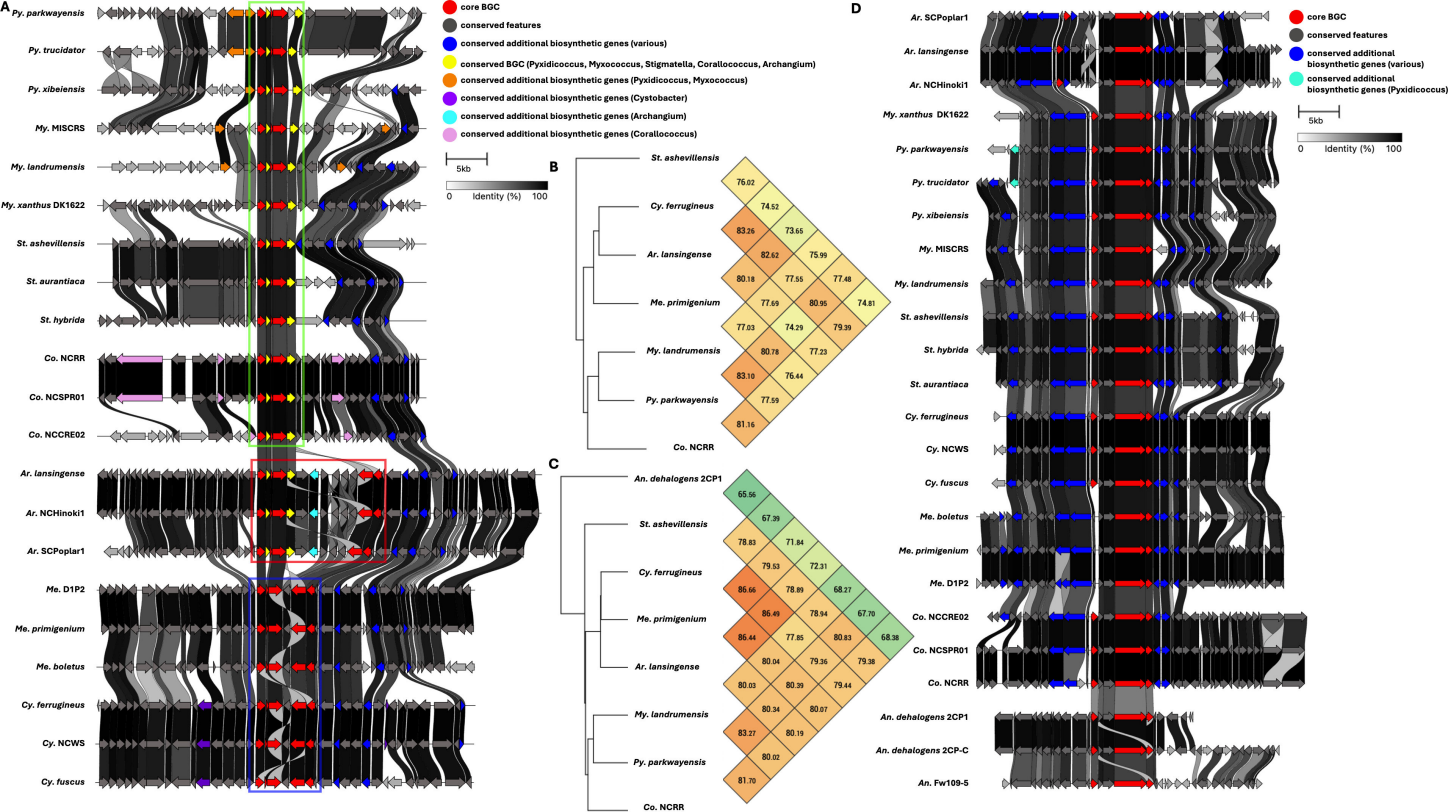
Conservation and genetic organization of the alkylpyrone BGC in Myxococcota (**A**) and ANI values for alkylpyrone BGCs across representatives of host genera (**B**). The discussed gene duplication event in *Archangium* (red box) and resulting differences in *Corallococcus*, *Pyxidicoccus*, *Myxococcus* (green box), and *Cystobacter* and *Melittangium* (blue box) clusters are indicated. Conservation and genetic organization of the VEPE/AEPE/TG-1 BGC in Myxococcota (**D**) and ANI values for VEPE/AEPE/TG-1 BGCs across representatives of host genera (**C**). AntiSMASH analysis provided .gbk files utilized to generate images in clinker and ANI values using OrthoANI (32, 36).

**Fig 5:**
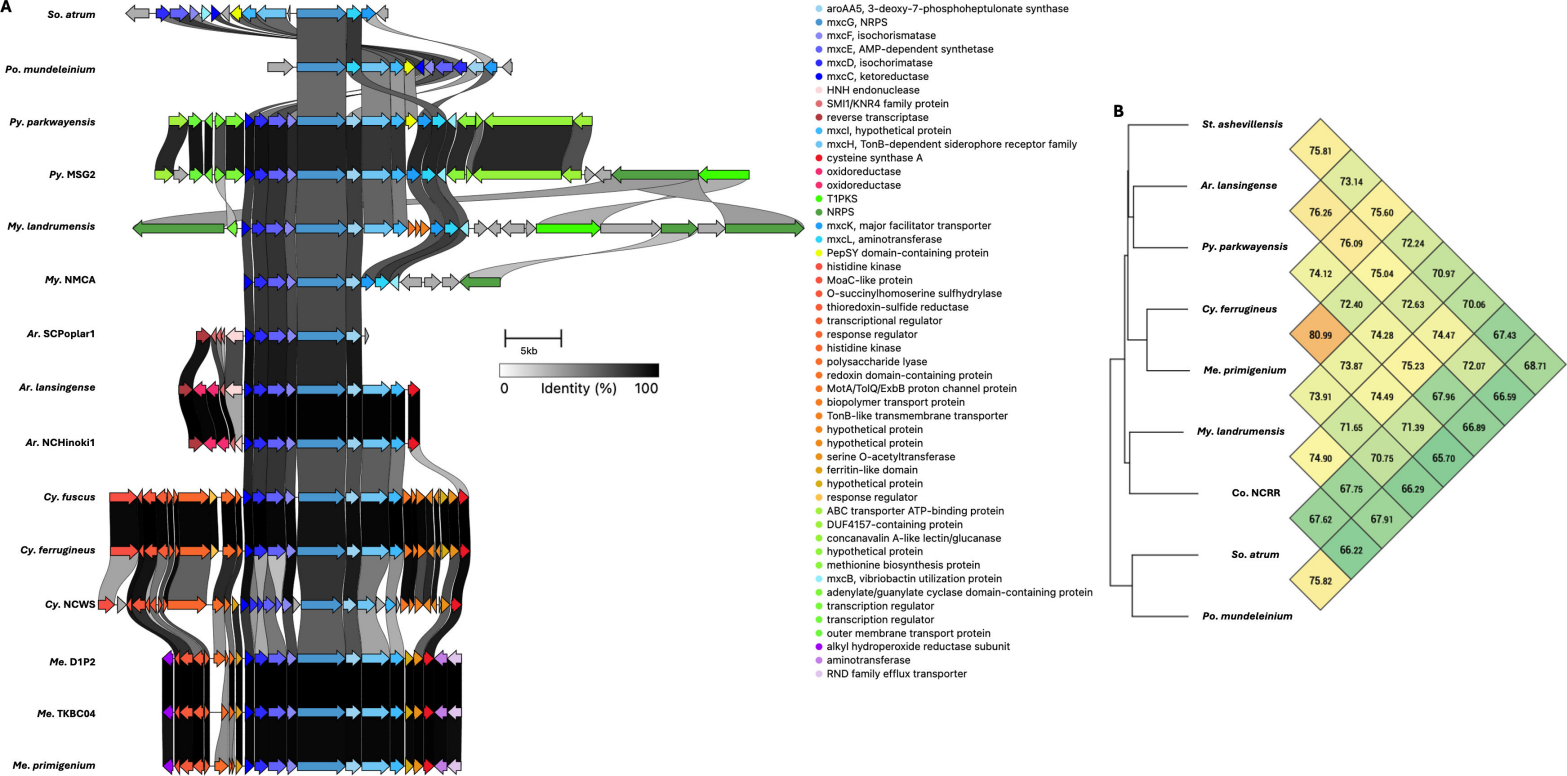
Conservation and genetic organization of the myxochelin BGC in Myxococcota (**A**) and ANI values for myxochelin BGCs across representatives of host genera (**B**). AntiSMASH analysis provided .gbk files utilized to generate images in clinker and ANI values using OrthoANI (32, 36).

Five BGCs are present in at least seven of the eleven genera analyzed (Table 2). All but one of these five BGCs can be unambiguously assigned using validated BGCs deposited in the MiBIG database, including the geosmin (BGC0001181), carotenoid (BGC0000648), VEPE/AEPE/TG-1 (BGC0000871), and myxochelin (BGC0002492) clusters (22–29). The remaining cluster has varying similarity (20%–63%) with a characterized alkylpyrone BGC (BGC0001831) and includes core type III polyketide synthase (PKS) features for alkylpyrone production (30). Notably, all five BGCs we identified to be conserved in the phylum are present in the model myxobacterium, *Myxococcus xanthus* DK1622. Maintenance of cluster organization and ANIs for each BGC was obtained using clinker and OAT, respectively, to assess the validity of our approach (Fig. 3–5) (31–33). This revealed shared identities for biosynthetic gene products and co-located gene products not anticipated to be directly involved in metabolite assembly. For example, recently discovered encapsulins thought to be involved in small, volatile terpene trafficking are consistently present in all geosmin clusters (34, 35). Maintained spatial organization of the five clusters can be observed at the genus level, and the organization of the VEPE/AEPE/TG-1 and carotenoid clusters is especially conserved in Myxococcia (Fig. 3 and 4). The VEPE/AEPE/TG-1 cluster is the only characterized cluster conserved in *Anaeromyxobacter*, and the geosmin cluster is the only characterized cluster conserved in *Nannocystis*. The VEPE/AEPE/TG-1 and alkylpyrone clusters are not conserved in any analyzed Polyangiia (*Nannocystis*, *Polyangium*, and *Sorangium*). Interestingly, our analysis suggests a gene duplication event apparent in the alkylpyrone BGCs from *Archangium* may have contributed to the differences in cluster organization in the Myxococcia (Fig. 4). The consistent presence, organization, and high identities between these five clusters are indicative of common ancestry and vertical inheritance in the phylum.

**TABLE 2:** Genus-level presence/absence of the five BGCs observed to be conserved in Myxococcota

BGC	Genera with	Genera without
Geosmin	*Archangium, Corallococcus, Cystobacter, Melittangium, Myxococcus, Nannocystis, Polyangium, Pyxidicoccus, Sorangium, Stigmatella*	*Anaeromyxobacter*
Carotenoid	*Archangium, Corallococcus, Cystobacter, Melittangium, Myxococcus, Polyangium, Pyxidicoccus, Sorangium, Stigmatella*	*Anaeromyxobacter, Nannocystis*
VEPE/AEPE/TG-1	*Anaeromyxobacter, Archangium, Corallococcus, Cystobacter, Melittangium, Myxococcus, Pyxidicoccus, Stigmatella*	*Nannocystis, Polyangium, Sorangium*
Alkylpyrone	*Archangium, Corallococcus, Cystobacter, Melittangium, Myxococcus, Pyxidicoccus, Stigmatella*	*Anaeromyxobacter, Nannocystis, Polyangium, Sorangium*
Myxochelin	*Archangium, Corallococcus, Cystobacter, Melittangium, Myxococcus, Pyxidicoccus, Polyangium, Stigmatella*	*Anaeromyxobacter, Nannocystis, Sorangium*

### Validation of identified BGCs conserved in myxobacteria

We sought to test the validity of our observation that the geosmin, carotenoid, VEPE/AEPE/TG-1, alkylpyrone, and myxochelin clusters are conserved in myxobacteria by determining the presence/absence of each BGC from lesser-studied genera not included in our pan-genome analysis due to a lack of sequenced representatives (Table 3). Notably, *Kofleria* and *Simulacricoccus* had no sequenced species to include in our analysis, and poor genome quality precluded the only sequenced *Plesiocystis*, *Plesiocystis pacifica* (37). At least two of the five conserved BGCs were present in all the myxobacteria included. All five BGCs were present in representative *Citreicoccus* and *Vitiosangium*. Representatives from *Labilithrix*, *Pseudenhygromyxa*, and *Vulgatibacter* host the fewest conserved clusters, with only two of the five BGCs present in each. However, it is worth noting that *Vulgatibacter incompetus* has just four BGCs in total, so the conserved VEPE/AEPE/TG-1 and alkylpyrone clusters present account for half of the BGCs in the genome. The myxochelin BGC was the least conserved cluster observed in the 11 analyzed myxobacteria (4/10), and the carotenoid and alkylpyrone BGCs were the most conserved (8/10).

**TABLE 3:** Validation of conserved BGCs using presence/absence data of representatives from genera not included in our pan-genome analysis^*a*^

Strain	Geosmin	Carotenoid	VEPE/AEPE/TG-1	Alkylpyrone	Myxochelin	Total
*Citreicoccus inhibens*	+	+	+	+	+	5/5
*Vitiosangium* sp. GDMCC 1.1324	+	+	+	+	+	5/5
*Chondromyces crocatus*	+	+	+	+	−	4/5
*Haliangium ochraceum*	+	+	+	−	+	4/5
*Aggregicoccus* sp. 17bor-14	−	+	+	+	−	3/5
*Enhygromyxa salina*	+	+	−	+	−	3/5
*Sandaracinus amylolyticus*	−	+	−	+	+	3/5
*Labilithrix luteola*	−	−	+	+	−	2/5
*Pseudenhygromyxa* sp. WMMC2535	+	+	−	−	−	2/5
*Vulgatibacter incompetus*	−	−	+	+	−	2/5
Total	7/11	9/11	8/11	9/11	5/11	

^*a*^ +, BGC present; −, BGC not present.

During the peer-review process, we were made aware that sequenced genomes for two additional *Hyalangium* species had been recently deposited, increasing the total number of sequenced strains to four. Fortuitously, this provided us an opportunity to further validate the conservation of the geosmin, carotenoid, VEPE/AEPE/TG-1, alkylpyrone, and myxochelin BGCs by determining their presence/absence in *Hyalangium*. AntiSMASH analysis of genome data from *Hyalangium gracile*, *Hyalangium minutum*, *Hyalangium rubrum*, and *Hyalangium versicolor* revealed genus-level conservation of all five BGCs (Table 4). Notably, the alkylpyrone cluster was not present in *H. minutum,* and *H. rubrum* does not possess a myxochelin cluster. Despite the highly fragmented assemblies for all sequenced *Hyalangium* with contig counts ranging from 43 to 104, our analysis of *Hyalangium* further validated the conservation of all five BGCs in Myxococcota.

**TABLE 4:** Validation of conserved BGCs using presence/absence data of recently sequenced *Hyalangium*^*a*^

Strain	Geosmin	Carotenoid	VEPE/AEPE/TG-1	Alkylpyrone	Myxochelin	Total
*Hyalangium gracile*	+	+	+	+	+	5/5
*Hyalangium minutum*	+	+	+	−	+	4/5
*Hyalangium rubrum*	+	+	+	+	−	4/5
*Hyalangium versicolor*	+	+	+	+	+	5/5
Total	4/4	4/4	4/4	3/4	3/4	

^*a*^ +, BGC present; −, BGC not present.

### Genus-level conservation of BGCs in myxobacteria

**Fig 6:**
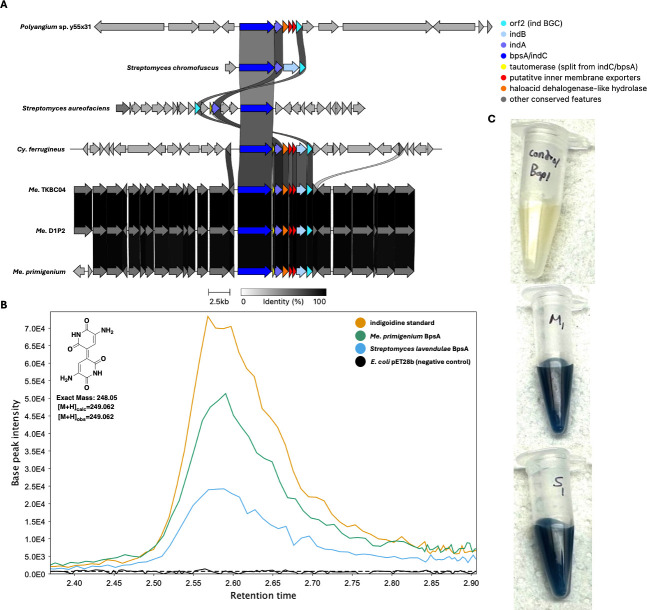
Conservation and genetic organization of the indigoidine BGC from *Str. chromofuscus*, *Str. aureofaciens*, and host Myxococcota (**A**). Extracted ion chromatograms (EICs) for 249-249.1 *m/z* obtained by LC-MS/MS analysis of an indigoidine standard, extracts from *E. coli* BAP1 expressing *bpsA* homologs from *Str. lavenduale* and *Me. primigenium,* and extracts from *E. coli* BAP1 with an empty plasmid as a negative control (**B**). Clarified culture broth from *E. coli* BAP1 (negative control; top), *E. coli* BAP1 expressing *bpsA* from *Me. primigenium* (middle), and *E. coli* BAP1 expressing *bpsA* from *Str. lavendulae* (bottom) (**C**). AntiSMASH analysis provided .gbk files for indigoidine clusters of Myxococcota (36). Indigoidine clusters for *Streptomyces* species were obtained from the MIBIG database as .gbk files. MZMine was used to generate EIC images (20).

Using our genus-level pan-genome data, we were also able to compare the distribution of BGCs across genera included in our data set (Table 5), so we sought to catalog the conserved BGCs per genus. The five BGCs conserved at the phylum level are the only characterized BGCs conserved in *Corallococcus* and *Cystobacter*. Beyond the five assigned BGCs conserved in the phylum, only two characterized BGCs are conserved across multiple genera. The myxoprincomide BGC (BGC0000393) is conserved in *Myxococcus* and *Pyxidicoccus* (38)*,* and the dkxanthene BGC (BGC0000986) is conserved in *Myxococcus* and *Stigmatella* (39). The indigoidine BGC (BGC0000727) from *Streptomyces aureofaciens* is conserved in *Melittangium* (40). The indigoidine BGC is also present in *Cystobacter ferrugineus* and *Polyangium* sp. y55x31. Shared identities with indigoidine clusters from *Str. aureofaciens* and *Streptomyces chromofuscus* (BGC0000375) suggest horizontal transfer of the cluster (Fig. 6A) (41). Excluding *Polyangium*, uncharacterized BGCs that encode for unknown metabolites are conserved in all genera (Table 5). Of these, a type 1 PKS cluster conserved in *Archangium* and *Melittangium* was the only unknown BGC present in multiple genera.

**TABLE 5:** Conserved characterized and uncharacterized BGCs (excluding BGCs included in Table 2) identified by pan-genome analysis, organized by genus^*a*^

Genus	Conserved characterized BGCs	Conserved unknown BGCs (by type)
*Anaeromyxobacter*	None	Betalactone thioamitide
*Archangium*	2-hydroxysorangiadenosine	**T1PKS** microviridin mixed hybrid (5)
*Corallococcus*	None	Hybrid NRPS-PKS mixed hybrid (3)
*Cystobacter^b^*	None	Mixed hybrid microviridin
*Melittangium^b^*	Indigoidine	Mixed hybrid **T1PKS**
*Myxococcus*	Myxochromide A **myxoprincomide** **dkxanthene**	Mixed hybrid hybrid NRPS-PKS (2)
*Pyxidicoccus*	**Myxoprincomide**	Mixed hybrid (2)
*Stigmatella^b^*	Myxothiazol aurachin A/D myxochromide S1 dawenol aurafuron **dkxanthene**	T1PKS mixed hybrid (3)
*Nannocystis*	Pyrronazol B 2-methyl-isoborneol	Phenazine
*Polyangium*	Sorangipyranone	None
*Sorangium*	Eremophilene	Microviridin

^*a*^ AntiSMASH-assigned cluster types were used to label uncharacterized BGCs with totals for each type included in parentheses when necessary. BGCs conserved in multiple genera are bolded.

^*b*^ Genera with fewer distinct species sequenced and lower confidence of cluster conservation.

### Heterologous expression of BpsA from *Me. primigenium*

After observing conservation of the indigoidine cluster in *Melittangium*, we sought to confirm similarity-based assignment of the cluster. An *indC*/*bpsA* homolog (WP_204491470.1) encoding the nonribosomal synthetase (NRPS), blue pigment synthetase A (BpsA), in the identified indigoidine BGC from *Me. primigenium* was heterologously expressed in *E. coli* BAP1. Numerous heterologous platforms for indigoidine production have been developed by expression of BpsA in various hosts (42–45). Comparing *E. coli* BAP1 expressing BpsA from *Me. primigenium* with *E. coli* BAP1 expressing BpsA from *Streptomyces lavendulae* (46, 47), an established indigoidine-producing strain (48, 49), we observed blue phenotypes indicative of indigoidine production from each strain (6C). Successful indigoidine production of *E. coli* BAP1 expressing *indC*/*bpsA* from *Me. primigenium* was confirmed by subsequent LC-MS/MS analysis of organic phase extracts from both *E. coli* strains, comparing indigoidine retention times, *m/z*, and fragmentation pattern (6B). These results confirm the similarity-based assignment and noted conservation of the indigoidine BGC in *Melittangium*.

## DISCUSSION

We identified five clusters using our pan-genome approach to identify BGCs conserved in myxobacteria. The high level of conservation and spatial organization found in each of the five conserved BGCs suggests vertical inheritance from a common ancestor. Vertical inheritance of these five clusters is also supported by variable ANIs for clusters present in extant lineages. Corroborating an inspirational pan-genome analysis of *Corallococcus* (11), we also found the majority of myxobacterial BGCs to be excluded from core pan-genomes for all analyzed genera. As noted, the low availability of representative, distinct species of *Cystobacter*, *Melittangium*, and *Stigmatella* offers a clear limitation in our approach. Validation of conservation for these five BGCs by determining their presence in available genome data of representative myxobacteria from genera not included in our pan-genome analysis revealed variable conservation for each cluster. These data support our conclusion that the geosmin, carotenoid, VEPE/AEPE/TG-1, myxochelin, and alkylpyrone clusters are broadly conserved in Myxococcota.

Excluding alkylpyrones, metabolites produced from all other conserved BGCs have characterized ecological functions. Geosmin is a ubiquitous, volatile sesquiterpenoid produced by numerous prokaryotes and eukaryotes (34). Geosmin producers repel predatory *Caenorhabditis elegans*, and geosmin has been proposed to serve as a non-toxic, chemical deterrent or aposematic signal (50). Carotenoids are lipophilic pigments produced by myxobacteria to quench toxic, reactive oxygen species (23, 25, 51). Iso-fatty acid signals produced by the VEPE/AEPE/TG-1 cluster are developmentally regulated signals that activate sporulation and fruiting body formation (27, 52, 53). Myxochelins are iron-chelating siderophores advantageous to iron competition in the environment (28, 29, 54–56). Considering the recent observation of myxobacterial interspecies conflict (57), competition for iron between myxobacteria in nature and the evolution of the myxochelin BGC could account for the reported variety of myxochelin analogs (56, 58–60). Notably, none of the metabolites with elucidated ecological roles are toxins such as antibacterials or antifungals; they instead have defensive, developmental, and metabolic roles. Although *in vitro* topoisomerase inhibition has been observed from alkylpyrones produced by *M. xanthus*, alkylpyrones and related alkylquinones and alkylresorcinols produced by type III PKSs have demonstrated a range of non-toxic activities such as alternative electron carriers in mycobacteria and membrane components that provide antibiotic resistance to *Streptomyces griseus* (30, 61, 62).

We suggest that the vertical inheritance of BGCs encoding for metabolites that broadly benefit myxobacterial resilience during environmental stress explains the conservation and maintenance of these clusters. We also suggest that the absence of broadly conserved toxins, often considered to benefit predation, supports specialization of clades and potential competition for nutrients in the phylum. The recently elucidated role of ambruticin in myxobacteria-myxobacteria competition provides an example of how specialization might benefit myxobacterial genera and species (57). The limited number of clusters conserved in the phylum also corroborates the reported correlation between taxonomic distance and metabolic profiles of myxobacteria (9). Conservation of the horizontally acquired indigoidine BGC in *Melittangium* offers insight into how gene transfer events could benefit specialization. Comparing the indigoidine clusters from *Str. chromofuscus* and *Str. aureofaciens*, the BpsA NRPS module includes a terminal tautomerase domain in the *Str. chromofuscus* cluster that is not present in BpsA from *Str. aureofaciens* (63). Instead, a discrete gene encoding for a homologous tautomerase is present in the indigoidine cluster from *Str. chromofuscus*. This observation suggests the evolution of two distinct indigoidine clusters that involved either excision of an NRPS-fused tautomerase or a fusion event between neighboring tautomerase and NRPS features. Mirroring these differences, the indigoidine BGC present in *Polyangium* sp. y55x31 includes a BpsA NRPS with a fused tautomerase, and the clusters from *Cystobacter* and *Melittangium* spp. have discrete tautomerase-encoding genes. Although parallel evolution of tautomerase fused/unfused indigoidine clusters in *Streptomyces* and myxobacteria cannot be ruled out, separate horizontal transfer events likely account for the differences in myxobacterial indigoidine BGCs. Our previous observation that the myxovirescin BGC conserved in *Myxococcus* spp. was acquired horizontally provides another example of a horizontal gene transfer event benefiting myxobacterial predation and specialization (16). Ultimately, our data and identification of conserved BGCs in Myxococcota reveal the vertical inheritance of biosynthetic pathways that produce metabolites involved in stress responses and chemical signaling, and the absence of BGCs encoding for toxic metabolites conserved at the phylum level supports competitive specialization of myxobacterial genera.

## MATERIALS AND METHODS

### Materials

*E. coli* DH5a and DH10B were used when cloning the indigoidine BGC, and *E. coli* BAP1 was utilized as a heterologous host for indigoidine production experiments. Cultivation of *E. coli* was done using lysogeny broth (LB) or LB agar. Myxobacteria were maintained on VY/4 plates (Baker’s yeast 2.5 g/L, CaCl_2_ × 2H_2_O 1.36 g/L, vitamin B_12_ 0.5 mg/L, and agar 15 g/L). Cycloheximide, nystatin, kanamycin, and isopropyl-β-D-1-thiogalactopyranoside (IPTG) were purchased from MilliporeSigma. Purified indigoidine to be used as an analytical standard was provided by Shreenika (San Diego, CA).

### Isolation of myxobacteria

Environmental isolates were obtained using our previously established methodology (16, 17). Standard prey-baiting methods using *E. coli* were used to isolate bacteriolytic myxobacteria. Air-dried soil samples were wetted with an antifungal solution (250 µg/mL of cycloheximide and nystatin) and pea-sized aliquots were plated on *E. coli* WAT plates. To prepare *E. coli* WAT plates, an *E. coli* lawn was grown overnight at 37°C and resuspended in 1 mL of antifungal solution. Subsequently, 300 µL of the solution was spread across a WAT agar (1.5% agar, 0.1% CaCl_2_) plate and air-dried to yield *E. coli* WAT plates. Soil-inoculated plates were incubated at 25°C for up to 4 weeks with daily checks for the appearance of lytic zones or fruiting bodies, and observed lytic zones or fruiting bodies were passaged to VY/4 plates repeatedly until monocultures were obtained. Filter paper methodology was used to isolate cellulolytic myxobacteria as previously described. Briefly, individual squares of autoclaved filter paper were placed on ST21 agar plates (1 g/L of K_2_HPO_4_, 20 mg/L of yeast extract, 14 g/L of agar, 1 g/L of KNO_3_, 1 g/L of MgSO_4_ × 7H_2_O, 1 g/L of CaCl_2_ × 2H_2_O, 0.1 g/L of MnSO_4_ × 7H_2_O, and 0.2 g/L of FeCl_3_), and aliquots of antifungal solution wetted soil were placed on filter paper edges. Soil-inoculated plates were incubated at 25°C for up to 2 months with bi-daily checks for growth starting after 2 weeks. Observed fruiting bodies were passaged to fresh ST21 filter paper plates until monocultures were obtained.

### Cultivation of isolates

All isolates were maintained on VY/4 plates and liquid cultures with CYH/2 media (0.75 g/L of casitone, 0.75 g/L of yeast extract, 2 g/L of starch, 0.5 g/L of soy flour, 0.5 g/L of glucose, 0.5 g/L of MgSO_4_·7H_2_O, 1 g/L of CaCl_2_·2H_2_O, 6 g/L of HEPES, 8 mg/L of EDTA-Fe, and 0.5 mg/L of vitamin B_12_).

### Sequencing methods

Monocultures of environmental isolates were utilized for isolation of genomic DNA using NucleoBond High Molecular Weight DNA Kits (Macherey-Nagel), and quality and concentration were assessed via Nanodrop spectrophotometry (Thermo Scientific NanoDrop One) and Qubit fluorometry (dsDNA HS Assay Kit, ThermoFisher Scientific). All isolates were sequenced using an Oxford Nanopore Minion flow cell (R10.4.1) and associated ligation sequencing kit, native barcoding kit, or rapid barcoding kit. Basecalling and demultiplexing were performed using Guppy (v6+), assembly was performed using Flye (v2.9.2+), and assembly error correction was performed using medaka (v1.11+) (64).  

### Pan-genome analysis

Sequenced myxobacterial isolates and FASTA files for all *Anaeromyxobacter, Archangium, Corallococcus, Cystobacter, Melittangium*, *Myxococcus*, *Nannocystis*, *Polyangium, Pyxidicoccus*, *Sorangium*, and *Stigmatella* genomes available at the NCBI Genome database were utilized for pan-genome analyses. All included myxobacteria can be found in Table S1. Following established methodology, all genomes were annotated with Prokka (v1.11) to provide GFF3 files for pan-genome analysis using Roary (v3.12.0) (65–67). Pan-genomes were obtained for each analyzed genera, and the resulting gene_presence_absence.csv files were utilized for BGC analysis.

### BGC analysis

AntiSMASH version 7.0 was used to annotate BGCs present in all analyzed myxobacterial genomes (36). Genes included in the resulting BGC GenBank files were subsequently used for comparison with gene presence/absence results from our pan-genome analysis to determine conserved genes within BGCs. For our analysis, we considered conserved BGCs from each genus to be clusters present in 50% of the analyzed genomes (per genus) that included ≥2 conserved genes. All conserved genes observed in each of the five conserved BGCs can be found in Tables S2 to S7. AntiSMASH analysis was also used for the validation of identified, conserved BGCs from genera not included in our pan-genome analysis. Generated GenBank files from antiSMASH were utilized as input in Clinker on the CAGECAT web server (v1.0) to produce BGC comparisons in s 2-5 (31, 33).

### Comparative genomics

OrthoANI calculations and tree generation were achieved using OAT (orthoANI tool v0.93.1) (32), and dDDH calculations and 16S rRNA gene sequence-based tree generation (Fig. S1) were performed on the type strain genome server website (68).

### Indigoidine BGC cloning

The presence of an *indC*/*bpsA* homolog (WP_204491470.1) within the chromosome of *Me. primigenium* ATCC 29037 was observed by antiSMASH analysis, and alignment of *bpsA* homologs from myxobacteria and *Streptomyces* spp. (Fig. S2) revealed further homology. Genomic DNA was extracted from *Me. primigenium* as previously described in the “Sequencing methods” section. Gene-specific primers were designed to amplify *indC*/*bpsA* from *Me. primigenium* and *Str. lavendulae,* including 20–40 bp overlaps homologous to the multiple cloning sites of pET-28a to enable seamless Gibson Assembly and Circular Polymerase Extension Cloning (CPEC) based cloning (69). Primer sequences used for amplification and cloning (listed in Table S8) were designed using SnapGene 7.1.1 and synthesized by Integrated DNA Technologies. PCR amplification of *indC*/*bpsA* from both *Me. primigenium* and *Str. lavendulae* was carried out using the TaKaRa PCR Amplification Kit. PCR products were purified using the GeneJET PCR Purification Kit (Thermo Fisher Scientific), and concentrations were determined via Nanodrop. Vector and insert DNA concentrations were normalized and combined at recommended molar ratios (2:1 insert to vector) according to the manufacturer’s protocol. Assembly of *indC*/*bpsA* from *Me. primigenium* into pET-28a was performed using Gibson Assembly (NEBuilder HiFi DNA Assembly Master Mix), generating construct pNS001. The control construct, pNS002, containing *indC*/*bpsA* from *Str. lavendulae*, was assembled via CPEC using Platinum SuperFi II PCR Master Mix. Assembled plasmids were introduced into either DH10B or DH5α *E. coli* strains. Transformants were selected on LB agar with kanamycin (50 µg/mL). Plasmids were isolated from overnight cultures using the ZymoPURE II Plasmid Prep Kit. Linearization was performed by restriction digestion for length verification, followed by analysis via gel electrophoresis. Sequence confirmation of pNS001 and pNS002 was performed by Plasmidsaurus. Verified pNS001 and pNS002 constructs (Table S9) were then transformed into *E. coli* BAP1 cells via electroporation for expression.

### Heterologous production of indigoidine

Single colonies of *E. coli* BAP1+pNS001 and *E. coli* BAP1+pNS001 were inoculated in LB broth with kanamycin (50 µg/mL) and grown at 30°C to an OD_600_ of 0.4 before induction with 200 mM IPTG. Cultures were incubated overnight at 18°C with shaking at 200 rpm. Indigoidine production was indicated by the development of blue pigmentation in liquid cultures. Following induction and cultivation, cultures were stored at −20°C until subsequent LC-MS/MS analysis. Aliquots (1.5 mL) of overnight *E. coli* cultures exhibiting blue pigmentation were vortexed and centrifuged at 15,000 × *g* for 5 min to pellet the cells along with insoluble pigment. The pellet was resuspended in 1:1 methanol:water, vortexed briefly, and centrifuged again under the same conditions. The supernatant was discarded, and the pellet was resuspended in dimethyl sulfoxide (DMSO) to extract indigoidine. A 100 µL aliquot of this extract was then diluted with 900 µL of DMSO, resulting in a 1:10 dilution. The diluted extract was transferred to autosampler vials for LC-MS/MS analysis. Extracts prepared as described above from cultures of *E. coli*+pET28b were prepared for LC-MS/MS analysis as negative controls.

### Mass spectrometry

Chromatographic and mass spectrometric analyses were performed using an Agilent 6530C quadrupole time-of-flight (Q-TOF) mass spectrometer equipped with a Dual Agilent Jet Stream electrospray ionization source, interfaced with an Agilent 1260 Infinity II HPLC system. Chromatographic separation was carried out using a Gemini 5 µm NX-C18 110 Å, 50 × 2.0 mm analytical column (Phenomenex) at a constant flow rate of 0.5 mL/min. Mobile phase A consisted of water with 0.1% formic acid, while mobile phase B consisted of methanol with 0.1% formic acid. The gradient program began with 90% A and 10% B, followed by a linear increase to 25% A and 75% B at minute 5. The composition was then increased linearly to 10% A and 90% B over 1 min and maintained at that mixture for an additional minute. Subsequently, the solvent composition was returned to the starting ratio over 1 min. To ensure system stability and readiness for subsequent injections, a 2 min re-equilibration phase was included. The total run time was set to 10 min, and the pressure limit was capped at 275 bar. A sample volume of 10 µL was injected per run. Injection draw and ejection speeds were set to 200 µL/min, and a needle height offset of 2 mm was used. A flush port needle wash (50% IPA/water) was enabled for 3 s, repeated three times. The autosampler was configured for sample overlap reduction with a sample flush-out factor of 5× the injection volume, though overlap injection mode was disabled for these runs. Data storage thresholds were set at 200 for MS and 5 for MS/MS, with centroid data collection enabled. The total cycle time was 2.3 s. The Q-TOF was operated in positive ionization mode with the following ion source conditions: gas temperature at 325°C, drying gas flow at 10 L/min, nebulizer pressure at 50 psi, sheath gas temperature at 350°C, and sheath gas flow at 12 L/min. Capillary voltage was set to 4,000 V, with nozzle voltage at 0 V. Fragmentor voltage was 200 V, skimmer at 65 V, and octopole RF set to 750 V. Data were collected in centroid mode with MS scans acquired across a mass range of 100–1,600 *m/z* at a rate of 5 spectra/s, and MS/MS scans across 50–1,305 *m/z* at a rate of 3 spectra/s. Transient times were set to 200 ms/spectrum for MS and 333.3 ms/spectrum for MS/MS, with 2,650 and 4,355 transients/spectrum, respectively. Collision energies for MS/MS were calculated using the equation (3 × *m/z*/100) + 15. The system automatically selected precursor ions with a maximum of six precursors per cycle, applying active exclusion after three MS/MS spectra were collected per ion within a 0.2 min window. Static exclusion windows were applied to exclude low- and high-mass ions (100–250 and 1,300–1,600 *m/z*), as well as known background ions, including *m/z* values of 922.0098, 531.40777, 553.38972, and 1083.791, with a delta tolerance of ±100 ppm. Precursor selection was based on abundance thresholds (10,000 counts, 0.01%) with iterative MS/MS enabled. Charge state preferences were set to prioritize 1+, 2+, and unknown precursors for fragmentation. Extracted ion chromatograms obtained from an indigoidine standard using the exact mass of indigoidine (249–249.1 *m/z*) were compared with extracts from *E. coli* BAP1 expressing *bpsA/indC* homologs from *Str. lavendulae* and *Me. primigenium* to confirm indigoidine production. An indigoidine standard was prepared at 1 mg/mL in DMSO and diluted 1:100 (final concentration 10 µg/mL); 2 µL (20 ng) of this working solution was injected for LC-MS/MS analysis under identical conditions as the samples.

## Data Availability

The data sets presented in this study can be found in online repositories. NCBI accession numbers are as follows: ASM4919486v1 (BB12-2); ASM4919212v1 (hickory4); ASM4906018v1 (MIELM); ASM4906006v1 (NCWS); ASM4906002v1 (PVMSAZ); ASM4905998v1 (NCHinoki1); CP185339 (UBH4); CP185340 (D1P2); ASM4286526v1 (SCPoplar1).

## SUPPLEMENTAL MATERIAL

Supplemental material aem.02151-25-s0001.pdf Tables S1 to S9; Fig. S1 to S4.

ASM does not own the copyrights to Supplemental Material that may be linked to, or accessed through, an article. The authors have granted ASM a non-exclusive, world-wide license to publish the Supplemental Material files. Please contact the corresponding author directly for reuse.
