# Supplementary material for: Establishing conserved biosynthetic gene clusters of the phylum Myxococcota
Source: Appl Environ Microbiol. 2025 Dec 11;92(1):e02151-25. doi: 10.1128/aem.02151-25 (PMC12766979; doi:10.1128/aem.02151-25)
Supplement: Supplemental material — Tables S1 to S9; Fig. S1 to S4. [file aem.02151-25-s0001.pdf]

Establishing Conserved Biosynthetic Gene Clusters of the Phylum Myxococcota

Shailaja Khanal, Nawal Shehata, Andrew Ahearne, Thomas Knehans, Constance B. Bailey, Paul D. Boudreau, and D. Cole Stevens

Supplemental Information

Table S1: Genomes included in pan-genome analysis.

Anaeromyxobacter	**Accession #**
An. dehalogens 2CP1	GCA_000022145.1
An. dehalogens 2CP-C	GCA_000013385.1
An. sp. Fw109-5	GCA_000017505.1
An. sp. K	GCA_000020805.1
An. oryzae Red232	GCA_023169945.1
An. paludicola Red630	GCA_023169965.1
An. soli SG29	GCA_022808855.1
An. diazotrophicus Red267	GCA_013340205.1
An. sp. PSR-1	GCA_000964525.1
An. sp. SG17	GCA_022690695.1
An. terrae SG22	GCA_022690685.1
An. sp. SG26	GCA_022690725.1
An. soli SG29	GCA_022808855.1
An. oryzisoli SG63	GCA_022690765.1
Archangium	
Ar. lipolyticum	GCA_024623785.1
Ar. sp. Cb G35	GCA_001896145.1
Ar. violaceum Cb vi76	GCA_003387095.1
Ar. gephyra DSM2261	GCA_003387095.1
Ar. gephyra DSM2261v2	GCA_001027285.1
Ar. lansingense	GCA_026626635.1
Ar. lansignense NCHinoki1	GCA_049059985.1
Ar. gephyra PVMSAZ	GCA_049060025.1
Ar. violaceum scpoplar1	GCA_042865265.1
Ar. violaceum SDU8	GCA_016887565.1
Ar. violaceum SDU34	GCA_016859125.1
Corallococcus	
Co. exiguus DSM14696	GCA_006376655.1
Co. sp. AB011P	GCA_003611605.1
Co. exiguus AB016	GCA_012985275.1
Co. sp. AB018	GCA_003986945.1
Co. sp. AB030	GCA_003668965.1

Co. exiguus AB031	GCA_012985225.1
Co. exiguus AB032A	GCA_013155175.1
Co sp. AB032C	GCA_003668935.1
Co. exiguus AB038A	GCA_013155195.1
Co. sp. AB038B	GCA_003668955.1
Co. exiguus AB039A	GCA_013155575.1
Co. excercitus AB043A	GCA_003611585.1
Co. excercitus AB043B	GCA_013116705.1
Co. sp. AB045	GCA_003668895.1
Co. interemptor AB047A	GCA_003668875.1
Co. sp. AB049A	GCA_003668885.1
Co. aberystwythensis AB050A	GCA_003612165.1
Co. exiguus AM006	GCA_013248865.1
Co. exiguus AM007	GCA_013248925.1
Co. sp. AS-1-12	GCA_020036995.1
Co. sp. AS-1-6	GCA_020037015.1
Co. macrosporus ATCC29039	GCA_017302985.1
Co. coralloides B035	GCA_004104415.1
Co. sp. BB11-1	GCA_026626625.1
Co. silvisoli C25j21	GCA_009909145.1
Co. praedator CA031B	GCA_003612125.1
Co. sp. CA031C	GCA_003612115.1
Co. sicarius CA040B	GCA_003611735.1
Co. sp. CA041A	GCA_003612075.1
Co. carmarthensis CA043D	GCA_003611695.1
Co. exercitus CA046A	GCA_013116615.1
Co. carmarthensis CA046B	GCA_013116625.1
Co. exiguus CA046D	GCA_013248915.1
Co. sp. CA047B	GCA_003612065.1
Co. exiguus CA048	GCA_012985235.1
Co. sp. CA049B	GCA_003611685.1
Co. llansteffanensis CA051B	GCA_003612055.1
Co. sp. CA053C	GCA_003611675.1
Co. terminator CA054A	GCA_003611635.1
Co. sp. CA054B	GCA_003611625.1
Co. exiguus DSM14696	GCA_009909105.1
Co. macrosporus DSM14697	GCA_002305895.1
Co. coralloides DSM2259	GCA_000255295.1
Co. sp. EGB	GCA_019968905.1

Co. macrosporus HW-1	GCA_000219105.1
Co. exiguus NCCRE002	GCA_017302975.1
Co. coralloides NCRR	GCA_026965535.1
Co. sp. NCSPR001	GCA_017309135.1
Co. sp. Z5C101001	GCA_007352635.1
Co. soli ZKHCc11396	GCA_014930455.1
Co. sp. bb12-1	GCA_026626765.1
Cystobacter	
Cy. ferrugineus Cbfe23	GCA_001887355.1
Cy. gracilis DSM14753	GCA_020103725.1
Cy. fuscus DSM2262	GCA_000335475.2
Cy. fuscus DSM52655	GCA_002305875.1
Cy. fuscus NCWS	GCA_049060065.1
Melittangium	
ATCC29037	GCA_016904885.1
D1P2	CP185340
DSM14713	GCA_002305855.1
TKBC04	-
Myxococcus	
My. sp. AB022	GCA_006547345.1
My. xanthus AB023	GCA_013116805.1
My. sp. AB025B	GCA_006518215.1
My. sp. AB036A	GCA_006547355.1
My. eversor AB053B	GCA_010894455.1
My. sp. AB056	GCA_006547365.1
My. xanthus AM003	GCA_013116825.1
My. xanthus AM005	GCA_013116865.1
My. sp. AM009	GCA_013372605.1
My. sp. AM010	GCA_013372585.1
My. sp. AM011	GCA_013372595.1
My. vastator AM301	GCA_010894475.1
My. llanfairpwllgwyngyllgogerychwyrndrobwllllantysiliogogogochensis AM401	GCA_006636215.1
My. sp. AS-1-15	GCA_020037055.1
My. xanthus ATCC27925	GCA_019895115.1
My. sp. BB12	ASM4919486v1
My. sp. CA005	GCA_006518205.1
My. sp. CA006	GCA_006518195.1
My. sp. CA010	GCA_006547325.1

My. sp. CA018	GCA_010998655.1
My. sp. CA023	GCA_010998615.1
My. sp. CA027	GCA_010279825.1
My. xanthus CA029	GCA_013116835.1
My. sp. CA033	GCA_013336625.1
My. sp. CA039A	GCA_013336645.1
My. sp. CA040A	GCA_013336725.1
My. sp. CA051A	GCA_013336705.1
My. sp. CA056	GCA_013336715.1
My. stipitatus CYD_1	GCA_021412625.1
My. xanthus DK101	GCA_025739225.1
My. xanthus DK1050	GCA_025739275.1
My. xanthus DK1622	GCA_000012685.1
DK1622_Tpase	GCA_015775755.1
My. stipitatus DSM14675	GCA_000331735.1
My. fulvus DSM16525	GCA_900111765.1
My. xanthus DSM16526	GCA_900106535.1
My. virescens DSM2260	GCA_900101905.1
My. xanthus DZ2(1)	GCA_000278585.2
My. xanthus DZ2(2)	GCA_018517205.1
My. xanthus DZ2(3)	GCA_020827275.1
My. xanthus DZF1	GCA_000340515.1
My. xanthus R31	GCA_016698685.1
My. xanthus MC359c15	GCA_006402735.1
My. xanthus MC335c16	GCA_006402415.1
My. xanthus KF4.3.9c1	GCA_006402015.1
My. xanthus GH3_5_6c2	GCA_006400955.1
My. xanthus GH5_1_9c20	GCA_006401215.1
My. fulvus Hickory4	GCA_049192125.1
My. dinghuensis K15C18031901	GCA_024198235.1
My. fulvus NBRC100333	GCA_007991095.1
My. virescens NBRC100334	GCA_007989405.1
My. qinghaiensis QH3KD-4-1	GCA_024198215.1
My. fulvus 11	GCA_023195975.1
My. guangdongensis K38C18041901	GCA_024198255.1
My. xanthus KF3_28c_11	GCA_006401635.1
My. hansupus	GCA_000280925.3
My. sp. MISCRS1	GCA_026626605.1
My. xanthus MxC21-1	GCA_032612255.1

My. sp. NMCA1	GCA_026810205.1
My. sp. RHSTA-1-4	GCA_020037125.1
My. landrumensis SCHIC003	GCA_017301635.1
My. sp. SDU36	GCA_030168875.1
My. sp. XM-1-1	GCA_020037095.1
Nannocystis	
N. exedens ATCC25963	GCA_900112715.1
N. bainbridgea BB15-2	GCA_028368995.1
Na. pusilla DSM53165	GCA_020073745.1
Na. exedens DSM71	GCA_002343915.1
Na. punicea FL3	GCA_026965555.1
Na. sp. ILAH1	GCA_026626585.1
Na. pusilla MIELM	GCA_049060185.1
Na. radixulma NCELM	GCA_028369095.1
Na. pusilla Na p29	GCA_026626665.1
Na. sp. RBIL2	GCA_026626745.1
Na. sp. SCPEA4	GCA_026626685.1
Na. sp. UBH4	CP185339
Polyangium	
Po. sp. 15x6	GCA_029960785.1
Po. sp. 6x1	GCA_029946515.1
Po. fumosum DSM14668	GCA_005144585.1
Po. sorediatum DSM14670	GCA_029946465.1
Po. spumosum DSM14734	GCA_009649845.1
Po. mundeleinium RJM3	GCA_028369105.1
Po. jinanense SDU13	GCA_028435265.1
Po. jinanense SDU14	GCA_028435365.1
Po. aurulentum SDU3-1	GCA_005144635.2
Po. sp. y55x31	GCA_029946505.1
Pyxidicoccus	
Py. caerfyrddinensis CA032A	GCA_010894405.1
Py. fallax CA059B	GCA_013155555.1
Py. trucidator CA060A	GCA_010894435.1
Py. fallax DSM14698	GCA_012933655.1
Py. sp. MSG2	GCA_026626705.1
Py. xibeiensis QH1ED-7-1	GCA_024198175.1
Py. parkwayensis SCPEA02	GCA_017301735.1
Sorangium	
So. cellulosum So0007-03	GCA_001589215.1

So. cellulosum So0008-312	GCA_001589285.1
So. cellulosum So0011-07	GCA_001589185.1
So. cellulosum So0149	GCA_001589205.1
So. cellulosum So0157-18	GCA_001589195.1
So. cellulosum So0157_2	GCA_000418325.1
So. cellulosum So0157_25	GCA_001589265.1
So. sp. Soce836	GCA_028553905.1
So. cellulosum Soce26	GCA_002950945.1
So. cellulosum Soce56	GCA_000067165.1
So. cellulosum Soce836	GCA_004135755.1
So. cellulosum SoceGT47	GCA_004135735.1
So. atrum wiwo2	GCA_028368935.1
Stigmatella	
St. hybrida DSM14722	GCA_020103775.1
St. erecta DSM16858	GCA_900111745.1
St. aurantiaca DSM17044	GCA_900109545.1
St. aurantica DW4_3-1(p)	GCA_000165485.1
St. aurantiaca DW4_3-1(c)	GCA_000168055.1
St. ashevillensis ncwal01	GCA_028368975.1

Table S2. Carotenoid BGC conserved features

carotenoid	conserved gene	# of strains	# in BGC
**Archangium**	Dehydrosqualene desaturase	12	12
	hypothetical protein	12	12
	All-trans-phytoene synthase	12	12
	Polyketide biosynthesis 3-hydroxy-3-methylglutaryl-ACP synthase PksG	12	12
	zeta-carotene-forming phytoene desaturase	12	12
	Serine/threonine-protein kinase PknL	12	12
	Sensor protein FixL	12	12
	Putative acetolactate synthase large subunit IlvB2	10	10
	S-(hydroxymethyl)glutathione dehydrogenase	12	9
	HTH-type transcriptional repressor YcgE	9	9
	Oxygen-independent coproporphyrinogen-III oxidase 1	9	9
	HTH-type transcriptional repressor YcgE	9	9
	Oxygen-independent coproporphyrinogen-III oxidase 1	9	9
	Serine/threonine-protein kinase Pkn1	9	9

	hypothetical protein	9	9
	dTDP-4-amino-4,6-dideoxy-D-glucose transaminase	9	9
	hypothetical protein	9	9
	hypothetical protein	9	9
	S-formylglutathione hydrolase YeiG	9	9
	HTH-type transcriptional regulator AcrR	9	9
	Putative fatty-acid--CoA ligase fadD21	9	9
	hypothetical protein	9	9
	hypothetical protein	8	7
	HTH-type transcriptional repressor YcgE	7	7
	hypothetical protein	8	8
	Acyclic carotenoid 1,2-hydratase	7	7
	hypothetical protein	7	6
	putative metallophosphoesterase	7	7
	NADH-quinone oxidoreductase subunit M	6	6
	Epimerase family protein	6	6
	hypothetical protein	9	9
	(2E,6E)-farnesyl diphosphate synthase	6	6
	Serine/threonine-protein kinase PknB	6	6
	HTH-type transcriptional regulator DmlR	6	6
	hypothetical protein	6	6
	hypothetical protein	6	6
	Na(+)/H(+) antiporter NhaG	6	6
	Methionine aminopeptidase 1, mitochondrial	6	6
	hypothetical protein	6	6
	hypothetical protein	6	6
	hypothetical protein	6	6
**Corallococcus**	zeta-carotene-forming phytoene desaturase	49	48
	All-trans-phytoene synthase	39	39
	Dehydrosqualene desaturase	39	39
	Acyclic carotenoid 1,2-hydratase	37	37
	hypothetical protein	37	37

	hypothetical protein	35	34
	hypothetical protein	49	47
	hypothetical protein	49	39
	Mercuric resistance operon regulatory protein	33	31
	HTH-type transcriptional repressor YcgE	34	26
	hypothetical protein	29	25
**Cystobacter**	Adenine deaminase	5	5
	hypothetical protein	5	5
	hypothetical protein	5	5
	Epimerase family protein	5	5
	hypothetical protein	5	5
	HTH-type transcriptional repressor YcgE	5	5
	hypothetical protein	5	5
	hypothetical protein	5	5
	Acyclic carotenoid 1,2-hydratase	5	5
	hypothetical protein	5	5
	Dehydrosqualene desaturase	5	5
	All-trans-phytoene synthase	5	5
	zeta-carotene-forming phytoene desaturase	5	5
	hypothetical protein	5	4
	putative metallophosphoesterase	5	4
	hypothetical protein	5	4
	Serine/threonine-protein kinase Pkn1	5	4
	hypothetical protein	3	3
**Melittangium**	zeta-carotene-forming phytoene desaturase	3	3
	All-trans-phytoene synthase	3	3
	NADPH-dependent 7-cyano-7-deazaguanine reductase	3	3
	Serine/threonine-protein kinase PrkC	3	3
	Guanosine-5'-triphosphate,3'-diphosphate pyrophosphatase	3	3
	hypothetical protein	3	3

**Myxococcus**	HTH-type transcriptional repressor YcgE	37	36
	HTH-type transcriptional repressor YcgE	38	37
	hypothetical protein	39	38
	hypothetical protein	39	38
	hypothetical protein	41	40
	hypothetical protein	38	37
	Acyclic carotenoid 1,2-hydratase	38	37
	Dehydrosqualene desaturase	39	38
	All-trans-phytoene synthase	39	38
	zeta-carotene-forming phytoene desaturase	39	38
	hypothetical protein	39	35
	Epimerase family protein	38	34
	Enterochelin esterase	39	35
	hypothetical protein	38	34
**Polyangium**	Spore protein SP21	9	9
	HTH-type transcriptional repressor YcgE	9	9
	Phytoene desaturase (lycopene-forming)	9	9
	Hydroxyneurosporene desaturase	9	9
	Farnesyl diphosphate synthase	9	9
	Alkaline phosphatase synthesis sensor protein PhoR	9	6
	hypothetical protein	8	6
	hypothetical protein	9	7
	Spore protein SP21	8	8
	hypothetical protein	8	8
	hypothetical protein	8	8
	hypothetical protein	8	8
	Acyclic carotenoid 1,2-hydratase	8	8
	RsbT co-antagonist protein RsbRD	9	9
	15-cis-phytoene synthase	8	8
**Pyxidicoccus**	Dehydrosqualene desaturase	6	5
	3-hydroxy-3-methylglutaryl-coenzyme A reductase	6	5

	hypothetical protein	7	6
	zeta-carotene-forming phytoene desaturase	7	7
	hypothetical protein	5	5
	Epimerase family protein	4	4
	Acyclic carotenoid 1,2-hydratase	4	4
	hypothetical protein	4	4
	HTH-type transcriptional repressor YcgE	4	4
	hypothetical protein	3	3
	All-trans-phytoene synthase	3	3
**Sorangium**	Phytoene desaturase (lycopene-forming)	14	8
	hypothetical protein	13	8
	Farnesyl diphosphate synthase	13	7
**Stigmatella**	Octaprenyl-diphosphate synthase	6	6
	zeta-carotene-forming phytoene desaturase	6	6
	Acyclic carotenoid 1,2-hydratase	6	6
	hypothetical protein	6	5
	Dehydrosqualene desaturase	3	3
	All-trans-phytoene synthase	3	3
	hypothetical protein	3	3
	Sensor protein ZraS	3	3
	putative HTH-type transcriptional regulator YybR	3	3
	putative metallophosphoesterase	3	3
	hypothetical protein	3	3
	Mercuric resistance operon regulatory protein	3	3
	Sensor protein ZraS	3	3
	Epimerase family protein	3	3
	hypothetical protein	3	3
	All-trans-phytoene synthase	3	3
	3-hydroxy-3-methylglutaryl-coenzyme A reductase	3	3
	Dehydrosqualene desaturase	3	3
	hypothetical protein	3	3

	hypothetical protein	3	3
	HTH-type transcriptional repressor YcgE	3	3
	Extracellular serine proteinase precursor	3	3

Table S3. Geosmin BGC conserved features

geosmin	conserved gene	# of strains	# in BGC
**Archangium**	Major membrane protein I	9	9
	Germacradienol/geosmin synthase	7	7
	Major membrane protein I	7	7
	hypothetical protein	9	6
	Protease HtpX	9	6
	N-acetylneuraminate epimerase	6	6
**Corallococcus**	Rod shape-determining protein MreB	50	25
	Undecaprenyl-phosphate mannosyltransferase	51	27
	L-2,4-diaminobutyrate decarboxylase	46	27
	2,3,4,5-tetrahydropyridine-2,6-dicarboxylate N-succinyltransferase	51	40
	Succinyl-diaminopimelate desuccinylase	49	40
	hypothetical protein	37	30
	Germacradienol/geosmin synthase	39	32
	Major membrane protein I	49	49
	Major membrane protein I	39	39
	hypothetical protein	25	25
	Cytochrome c-type protein NrfH	28	27
	Cytochrome c-552 precursor	31	28
	hypothetical protein	36	33
**Cystobacter**	4'-demethylrebeccamycin synthase	5	5
	Germacradienol/geosmin synthase	5	5
	hypothetical protein	5	5
	Major membrane protein I	5	5
	Major membrane protein I	5	5
	UDP-glucose 6-dehydrogenase TuaD	5	5

	Serine/threonine-protein kinase StkP	5	3
	Multidrug resistance protein 3	4	4
	hypothetical protein	3	3
**Melittangium**	zeta-carotene-forming phytoene desaturase	3	3
	All-trans-phytoene synthase	3	3
	NADPH-dependent 7-cyano-7-deazaguanine reductase	3	3
	Serine/threonine-protein kinase PrkC	3	3
	Guanosine-5'-triphosphate,3'-diphosphate pyrophosphatase	3	3
	hypothetical protein	3	3
**Myxococcus**	Germacradienol/geosmin synthase	36	35
	Transposon Tn10 TetC protein	39	34
	hypothetical protein	39	35
	hypothetical protein	38	34
**Nannocystis**	Major membrane protein I	12	11
	Germacradienol/geosmin synthase	11	11
	Major membrane protein I	9	8
	putative MscS family protein YkuT	8	7
	Minor extracellular protease Epr precursor	7	6
**Polyangium**	Major membrane protein I	9	9
	Germacradienol/geosmin synthase	8	8
	L-glyceraldehyde 3-phosphate reductase	8	8
	HTH-type transcriptional repressor ComR	8	8
	Major membrane protein I	8	8
**Pyxidicoccus**	Germacradienol/geosmin synthase	5	5
	Major membrane protein I	5	5
	Major membrane protein I	5	5
	putative oxidoreductase	6	3

**Sorangium**	Germacradienol/geosmin synthase	8	8
	Major membrane protein I	8	8
	Major membrane protein I	8	8
	putative MscS family protein YkuT	7	7
**Stigmatella**	Spermidine synthase	6	4
	hypothetical protein	6	4
	hypothetical protein	6	4
	NADP-dependent alcohol dehydrogenase C 2	3	3
	Major membrane protein I	3	3
	hypothetical protein	3	3
	F420-dependent glucose-6-phosphate dehydrogenase	3	3
	Serine/threonine-protein kinase pkn6	3	3
	hypothetical protein	3	3
	hypothetical protein	3	3
	HTH-type transcriptional regulator DmlR	3	3
	Major membrane protein I	3	3
	Germacradienol/geosmin synthase	3	3
	Major membrane protein I	3	3
	Germacradienol/geosmin synthase	3	3
	Phosphatidylglycerol lysyltransferase	3	3
	Sporulation initiation phosphotransferase F	3	3
	Major membrane protein I	3	3

Table S4. VEPE/AEPE/TG-1 BGC conserved features

VEPE/AEPE/TG-1	conserved gene	# of strains	# in BGC
**Anaeromyxobacter**			
**Archangium**	Threonylcarbamoyl-AMP synthase	12	6
	Protein ApaG	12	12
	Glycogen debranching enzyme	12	12
	hypothetical protein	12	12
	Phosphoserine phosphatase	12	12
	hypothetical protein	12	12

	Long-chain-fatty-acid--CoA ligase	12	12
	3 beta-hydroxysteroid dehydrogenase/Delta 5-->4-isomerase	12	12
	Response regulator PleD	12	10
	Glucose-1-phosphate adenylyltransferase	12	11
	Aminodeoxyfutalosine deaminase	12	11
	RNA-splicing ligase RtcB	11	9
	Ferrochelatase	10	10
	1D-myo-inositol 2-acetamido-2-deoxy-alpha-D-glucopyranoside deacetylase	7	7
	Maltooligosyl trehalose synthase	7	7
	Inner membrane protein alx	9	9
	Putative ketoacyl reductase	7	7
	hypothetical protein	6	6
	3 beta-hydroxysteroid dehydrogenase/Delta 5-->4-isomerase	6	6
**Corallococcus**	Outer membrane protein assembly factor BamB precursor	49	34
	Aminodeoxyfutalosine deaminase	51	41
	Glucose-1-phosphate adenylyltransferase	51	41
	hypothetical protein	51	42
	Phosphoserine phosphatase	49	36
	Glycogen debranching enzyme	49	25
	Protein ApaG	49	25
	Putative niacin/nicotinamide transporter NaiP	39	29
	hypothetical protein	36	27
	3 beta-hydroxysteroid dehydrogenase/Delta 5-->4-isomerase	49	38
	Long-chain-fatty-acid--CoA ligase	40	40
	3 beta-hydroxysteroid dehydrogenase/Delta 5-->4-isomerase	49	28
	Glycogen debranching enzyme	49	25
**Cystobacter**	Response regulator rcp1	5	5
	Glucose-1-phosphate adenylyltransferase	5	5
	hypothetical protein	5	5
	hypothetical protein	5	5

	Phosphoserine phosphatase	5	5
	Ferrochelatase	5	5
	Bacteriophytochrome	5	5
	hypothetical protein	5	5
	ATP-dependent DNA helicase PcrA	5	5
	3 beta-hydroxysteroid dehydrogenase/Delta 5-->4-isomerase	5	5
	Glycogen debranching enzyme	5	5
	Inner membrane protein alx	5	5
	hypothetical protein	5	5
	hypothetical protein	5	5
	hypothetical protein	5	5
	hypothetical protein	5	5
	Multidrug resistance protein NorM	5	5
	Aminodeoxyfutalosine deaminase	5	5
	Glucose 1-dehydrogenase 4	5	5
	Response regulator PleD	5	5
	Maltooligosyl trehalose synthase	5	5
	Protein ApaG	5	5
	Threonylcarbamoyl-AMP synthase	5	5
	Epimerase family protein	5	5
	hypothetical protein	5	5
	3 beta-hydroxysteroid dehydrogenase/Delta 5-->4-isomerase	5	5
	Long-chain-fatty-acid--CoA ligase	5	5
	Bis(5'-nucleosyl)-tetraphosphatase PrpE [asymmetrical]	4	4
**Melittangium**	Phosphoserine phosphatase	4	3
	Response regulator PleD	4	3
	Glycogen debranching enzyme	4	3
	Threonylcarbamoyl-AMP synthase	4	3
	Protein ApaG	4	3
	Inner membrane protein alx	4	3
	hypothetical protein	4	3
	Long-chain-fatty-acid--CoA ligase	4	3

	3 beta-hydroxysteroid dehydrogenase/Delta 5-->4-isomerase	4	3
	Glucose-1-phosphate adenylyltransferase	4	3
	Aminodeoxyfutalosine deaminase	4	3
	ATP-dependent DNA helicase PcrA	4	3
	hypothetical protein	4	3
	Response regulator rcp1	4	3
**Myxococcus**	Ferrochelatase	39	37
	Protein ApaG	62	59
	Inner membrane protein alx	39	37
	Maltooligosyl trehalose synthase	39	37
	Glycogen debranching enzyme	39	37
	Phosphoserine phosphatase	62	61
	Long-chain-fatty-acid--CoA ligase	39	37
	hypothetical protein	63	61
	Levodione reductase	39	37
	Response regulator PleD	62	59
	3 beta-hydroxysteroid dehydrogenase/Delta 5-->4-isomerase	39	36
	GDP-6-deoxy-D-mannose reductase	38	36
	hypothetical protein	38	36
	RNA-splicing ligase RtcB	38	37
	hypothetical protein	38	35
	Outer membrane protein assembly factor BamB precursor	38	35
	hypothetical protein	62	50
	hypothetical protein	38	36
	putative N-acetyltransferase YafP	37	34
	hypothetical protein	62	52
	Aminodeoxyfutalosine deaminase	63	60
	hypothetical protein	63	61
	Glucose-1-phosphate adenylyltransferase	63	60
**Pyxidicoccus**	3 beta-hydroxysteroid dehydrogenase/Delta 5-->4-isomerase	7	7
	GDP-6-deoxy-D-mannose reductase	7	7

	Long-chain-fatty-acid--CoA ligase	7	7
	hypothetical protein	7	7
	Phosphoserine phosphatase	7	7
	Glycogen debranching enzyme	7	6
	hypothetical protein	7	6
	Protein ApaG	7	5
	Glucose-1-phosphate adenylyltransferase	7	5
	RNA-splicing ligase RtcB	7	5
	hypothetical protein	7	5
	Response regulator PleD	7	5
	Aminodeoxyfutalosine deaminase	7	5
	hypothetical protein	5	5
	4-formylbenzenesulfonate dehydrogenase TsaC1/TsaC2	5	5
	ATP-dependent DNA helicase PcrA	5	5
	hypothetical protein	7	4
	Maltooligosyl trehalose synthase	4	4
	hypothetical protein	4	4
	putative peptidase	3	3
	Ferrochelatase	3	3
	Inner membrane protein alx	3	3
	hypothetical protein	3	3
	Threonylcarbamoyl-AMP synthase	7	3
**Stigmatella**	3-oxoacyl-[acyl-carrier-protein] reductase FabG	6	6
	Glycogen debranching enzyme	6	6
	Inner membrane protein alx	6	6
	Ferrochelatase	6	6
	Threonylcarbamoyl-AMP synthase	6	6
	hypothetical protein	6	6
	hypothetical protein	6	6
	hypothetical protein	6	6
	ATP-dependent DNA helicase PcrA	6	6
	Glucose-1-phosphate adenylyltransferase	6	6

	3 beta-hydroxysteroid dehydrogenase/Delta 5-->4-isomerase	6	6
	Long-chain-fatty-acid--CoA ligase	6	6
	Protein ApaG	6	6
	Aminodeoxyfutalosine deaminase	6	6
	Response regulator PleD	6	6
	hypothetical protein	6	6
	Phosphoserine phosphatase	6	6
	putative ABC transporter ATP-binding protein YxlF	6	6
	hypothetical protein	6	6
	hypothetical protein	6	6
	putative sensor histidine kinase TcrY	3	3
	hypothetical protein	3	3
	Metallo-beta-lactamase L1 precursor	3	3
	hypothetical protein	3	3
	hypothetical protein	3	3
	hypothetical protein	3	3
	Membrane-bound lytic murein transglycosylase A precursor	3	3
	Maltooligosyl trehalose synthase	3	3
	Epimerase family protein	3	3
	hypothetical protein	3	3
	hypothetical protein	6	3
	Membrane-bound lytic murein transglycosylase A precursor	3	3
	Hydroxyacylglutathione hydrolase	3	3
	Maltooligosyl trehalose synthase	3	3
	Epimerase family protein	3	3

Table S5. Myxochelin BGC conserved features

**myxochelin**	**conserved gene**	**# of strains**	**# in BGC**
**Archangium**	2,3-dihydro-2,3-dihydroxybenzoate dehydrogenase	11	11
	hypothetical protein	12	9
	hypothetical protein	11	9
	hypothetical protein	9	9
	hypothetical protein	9	9

	hypothetical protein	10	8
	hypothetical protein	8	8
	hypothetical protein	8	8
	Phospho-2-dehydro-3-deoxyheptonate aldolase	8	8
	HTH-type transcriptional regulator TtgR	9	7
	Putative ribosome biogenesis GTPase RsgA	7	6
	2,3-dihydroxybenzoate-AMP ligase	6	6
	Isochorismate synthase DhbC	6	6
	hypothetical protein	6	6
	Dimodular nonribosomal peptide synthase	6	6
	hypothetical protein	6	6
	Isochorismatase	6	6
	Pentalenene oxygenase	6	6
**Corallococcus**	hypothetical protein	39	26
	Phospho-2-dehydro-3-deoxyheptonate aldolase	46	45
	Dimodular nonribosomal peptide synthase	36	35
	Isochorismatase	35	32
	2,3-dihydroxybenzoate-AMP ligase	44	42
	Isochorismate synthase DhbC	36	32
	2,3-dihydro-2,3-dihydroxybenzoate dehydrogenase	36	31
	3-aminobutyryl-CoA aminotransferase	48	40
	Purine efflux pump PbuE	36	30
**Cystobacter**	Biopolymer transport protein ExbB	5	5
	cAMP receptor protein	5	5
	HTH-type transcriptional repressor KstR2	5	5
	3-oxoadipate CoA-transferase subunit A	5	5
	Dimodular nonribosomal peptide synthase	5	5
	2,3-dihydroxybenzoate-AMP ligase	5	5
	Serine acetyltransferase	5	5
	hypothetical protein	5	5
	Phospho-2-dehydro-3-deoxyheptonate aldolase	5	5

	Isochorismatase	5	5
	Isochorismate synthase DhbC	5	5
	2,3-dihydro-2,3-dihydroxybenzoate dehydrogenase	5	5
	Thiol-disulfide oxidoreductase ResA	5	5
	hypothetical protein	5	5
	Thioredoxin reductase	5	5
	O-succinylhomoserine sulfhydrylase	5	5
	Cyclic pyranopterin monophosphate synthase accessory protein 2	5	5
	Cysteine synthase	5	5
	Biopolymer transport protein ExbD	5	5
	Alkaline phosphatase synthesis transcriptional regulatory protein PhoP	5	5
	3-oxoadipate CoA-transferase subunit B	5	4
	Alcohol dehydrogenase	5	4
	hypothetical protein	4	4
	hypothetical protein	4	4
	hypothetical protein	4	4
	hypothetical protein	4	4
	hypothetical protein	4	4
	Vitamin B12 transporter BtuB precursor	4	4
	6-phosphogluconolactonase	5	3
	hypothetical protein	5	3
	Polyketide biosynthesis 3-hydroxy-3-methylglutaryl-ACP synthase PksG	4	3
	hypothetical protein	4	3
	Sensor protein kinase WalK	4	4
	hypothetical protein	3	3
	hypothetical protein	3	3
**Melittangium**	50S ribosomal protein L13	4	4
	30S ribosomal protein S9	4	4
	FHA domain-containing protein FhaB	4	4
	Selenide, water dikinase	4	4
	Ribonuclease PH	4	4

	hypothetical protein	4	3
	Twitching mobility protein	4	3
	ATP-dependent DNA helicase PcrA	4	3
	hypothetical protein	4	3
	hypothetical protein	4	3
	hypothetical protein	4	3
	hypothetical protein	4	3
	hypothetical protein	4	3
	Mannan endo-1,4-beta-mannosidase precursor	4	3
	NTE family protein RssA	4	3
	hypothetical protein	3	3
	hypothetical protein	3	3
	Regulatory protein RecX	3	3
	Outer membrane protein assembly factor BamD precursor	3	3
	N-acetylmuramoyl-L-alanine amidase AmiC precursor	3	3
	Non-canonical purine NTP pyrophosphatase	3	3
	hypothetical protein	3	3
	hypothetical protein	3	3
	Aminopeptidase S	3	3
	hypothetical protein	3	3
	hypothetical protein	3	3
	ABC transporter ATP-binding protein YojI	3	3
	Potassium-transporting ATPase A chain	3	3
	Potassium-transporting ATPase B chain	3	3
	Sensor protein KdpD	3	3
	hypothetical protein	3	3
	Biotin biosynthesis cytochrome P450	3	3
	hypothetical protein	3	3
	Demethylrebeccamycin-D-glucose O-methyltransferase	3	3
	NADP-dependent alcohol dehydrogenase C 2	3	3
	hypothetical protein	3	3
	Linear gramicidin synthase subunit D	3	3
	cAMP receptor protein	3	3

	hypothetical protein	3	3
	Tyrocidine synthase 3	3	3
	Pentalenene oxygenase	3	3
	hypothetical protein	3	3
	Potassium-transporting ATPase C chain	3	3
	Alginate biosynthesis sensor protein KinB	3	3
	hypothetical protein	3	3
	Polyketide synthase PksL	3	3
	Polyketide biosynthesis protein PksE	3	3
	Pentachlorophenol 4-monooxygenase	3	3
	Polyketide synthase PksJ	3	3
	hypothetical protein	3	3
	hypothetical protein	3	3
	Serine/threonine-protein kinase Pkn1	3	3
	Polyketide biosynthesis 3-hydroxy-3-methylglutaryl-ACP synthase PksG	3	3
	Alcohol dehydrogenase	3	3
	3-oxoadipate CoA-transferase subunit B	3	3
	3-oxoadipate CoA-transferase subunit A	3	3
	HTH-type transcriptional repressor KstR2	3	3
	Cyclic pyranopterin monophosphate synthase accessory protein 2	3	3
	DNA protection during starvation protein	3	3
	Isochorismate synthase DhbC	3	3
	Serine acetyltransferase	3	3
	Cysteine synthase	3	3
	Prolyl tripeptidyl peptidase precursor	3	3
	hypothetical protein	3	3
**Myxococcus**	hypothetical protein	48	35
	Macrolide export ATP-binding/permease protein MacB	38	36
	2,3-dihydro-2,3-dihydroxybenzoate dehydrogenase	38	37
	Isochorismate synthase DhbC	38	37
	2,3-dihydroxybenzoate-AMP ligase	38	37

	Isochorismatase	38	37
	Dimodular nonribosomal peptide synthase	38	37
	Phospho-2-dehydro-3-deoxyheptonate aldolase	38	37
	Hexuronate transporter	38	37
	3-aminobutyryl-CoA aminotransferase	38	37
	Vibriobactin utilization protein ViuB	38	37
	hypothetical protein	38	37
	hypothetical protein	38	33
	Limonene 1,2-monooxygenase	38	33
	Phthiocerol/phenolphthiocerol synthesis polyketide synthase type I PpsE	37	32
	Phthiocerol/phenolphthiocerol synthesis polyketide synthase type I PpsE	37	32
	Glycogen synthase	38	32
	Linear gramicidin dehydrogenase LgrE	38	32
	hypothetical protein	38	32
	hypothetical protein	38	32
	hypothetical protein	38	32
	hypothetical protein	37	32
	hypothetical protein	38	32
	Phthiocerol synthesis polyketide synthase type I PpsE	36	32
**Polyangium**	Vitamin B12 transporter BtuB precursor	7	7
	hypothetical protein	7	7
	Glutamate-1-semialdehyde 2,1-aminomutase	7	7
	Phospho-2-dehydro-3-deoxyheptonate aldolase	5	5
**Pyxidicoccus**	cAMP receptor protein	7	5
	hypothetical protein	7	4
	Bifunctional protein PaaZ	6	4
	Isochorismate synthase DhbC	4	4
	2,3-dihydro-2,3-dihydroxybenzoate dehydrogenase	4	4
	Vibriobactin utilization protein ViuB	3	3
	D-alanine--D-alanine ligase	4	3

	Phospho-2-dehydro-3-deoxyheptonate aldolase	3	3
	2,3-dihydroxybenzoate-AMP ligase	3	3
	Adenylate cyclase 2	3	3
	Putative outer membrane protein precursor	3	3
	Putative pyridoxal phosphate-dependent acyltransferase	3	3
	Phthiocerol synthesis polyketide synthase type I PpsC	3	3
	Heptaprenyl diphosphate synthase component 2	7	3
	Biopolymer transport protein ExbB	4	4
	Quaternary ammonium compound-resistance protein SugE	7	3
	Phthiocerol synthesis polyketide synthase type I PpsE	3	3
**Stigmatella**	Isochorismatase	6	6
	Isochorismate synthase DhbC	6	6
	2,3-dihydroxybenzoate-AMP ligase	6	6
	NADPH-dependent ferric-chelate reductase	6	6
	Transcription elongation factor GreB	6	6
	PhoH-like protein	6	5
	Biotin biosynthesis cytochrome P450	6	5
	Hemin transport system permease protein HmuU	6	5
	3-aminobutyryl-CoA aminotransferase	6	5
	Phospho-2-dehydro-3-deoxyheptonate aldolase	6	5
	hypothetical protein	6	5
	ATP-dependent RNA helicase HrpB	4	3
	hypothetical protein	3	3
	hypothetical protein	3	3
	Purine efflux pump PbuE	3	3
	Vitamin B12 transporter BtuB precursor	3	3
	2,3-dihydro-2,3-dihydroxybenzoate dehydrogenase	3	3
	Pyridoxal 4-dehydrogenase	3	3
	hypothetical protein	3	3
	Trans-aconitate 2-methyltransferase	3	3
	hypothetical protein	3	3
	Hemin-binding periplasmic protein HmuT precursor	3	3

	Hemin import ATP-binding protein HmuV	3	3
	hypothetical protein	3	3
	Dimodular nonribosomal peptide synthase	3	3
	Phthiotriol/phenolphthiotriol dimycocerosates methyltransferase	3	3
	hypothetical protein	3	3
	Peptide methionine sulfoxide reductase MsrB	3	3
	hypothetical protein	3	3
	hypothetical protein	6	4
	lipid kinase YegS	6	4
	putative oxidoreductase	6	4
	Chaperone protein DnaK	6	4
	Dimodular nonribosomal peptide synthase	3	3
	2,3-dihydro-2,3-dihydroxybenzoate dehydrogenase	3	3
	hypothetical protein	3	3
	3-isopropylmalate dehydrogenase	3	3
	3-isopropylmalate dehydratase small subunit	3	3
	3-isopropylmalate dehydratase large subunit	3	3
	2-isopropylmalate synthase	3	3
	hypothetical protein	3	3
	Ubiquinone/menaquinone biosynthesis C-methyltransferase UbiE	6	3
	hypothetical protein	4	3

Table S6. Alkylpyrone BGC conserved features

alkylpyrone	conserved gene	# of strains	# in BGC
**Archangium**	Putative peroxiredoxin bcp	11	11
	Response regulator SaeR	12	11
	2-octaprenyl-3-methyl-6-methoxy-1,4-benzoquinol hydroxylase	12	11
	Alpha-pyrone synthesis polyketide synthase-like Pks11	12	11
	hypothetical protein	12	11
	Multifunctional cyclase-dehydratase-3-O-methyl transferase TcmN	12	11
	hypothetical protein	12	10
	putative lipoprotein YbbD precursor	12	10
	hypothetical protein	10	9

	putative oxidoreductase	12	9
	hypothetical protein	9	9
	Acyl carrier protein	9	9
	hypothetical protein	9	9
	Putative ligase	9	9
	Alpha-pyrone synthesis polyketide synthase-like Pks18	9	9
	Methyl-accepting chemotaxis protein CtpH	9	9
	Carbonic anhydrase 1	9	8
	hypothetical protein	9	8
	Bicarbonate transporter BicA	9	8
	hypothetical protein	8	7
	Long-chain-fatty-acid--AMP ligase FadD26	7	7
	Decaprenyl-phosphate phosphoribosyltransferase	7	7
	hypothetical protein	7	7
	Luminescence regulatory protein LuxO	7	6
	hypothetical protein	6	6
	Cocaine esterase	6	6
	Meromycolate extension acyl carrier protein	6	6
	hypothetical protein	6	6
	Alkaline phosphatase synthesis transcriptional regulatory protein PhoP	6	6
	hypothetical protein	6	6
**Corallococcus**	Alpha-pyrone synthesis polyketide synthase-like Pks11	49	30
	hypothetical protein	49	30
	Long-chain-fatty-acid--AMP ligase FadD26	49	30
	hypothetical protein	46	30
	hypothetical protein	47	28
	hypothetical protein	41	27
	hypothetical protein	39	27
	hypothetical protein	39	26
	Multifunctional cyclase-dehydratase-3-O-methyl transferase TcmN	49	28
	putative transcriptional regulatory protein TcrX	49	27

	hypothetical protein	49	25
**Cystobacter**	Proline--tRNA ligase	5	4
	Alpha-pyrone synthesis polyketide synthase-like Pks18	5	4
	hypothetical protein	5	4
	Beta-glucanase precursor	5	4
	Putative trans-acting enoyl reductase	5	4
	Methyl-accepting chemotaxis protein CtpH	5	4
	Long-chain-fatty-acid--AMP ligase FadD29	5	4
	Putative ligase/MSMEI_5285	5	4
	High-affinity branched-chain amino acid transport ATP-binding protein LivF	5	4
	hypothetical protein	5	4
	hypothetical protein	5	4
	Endonuclease YhcR precursor	5	4
	hypothetical protein	5	4
	Alpha-pyrone synthesis polyketide synthase-like Pks11	5	4
	Acyl carrier protein	5	4
	Acyl carrier protein	5	4
	putative decaprenylphosphoryl-beta-D-ribose oxidase	5	4
	Decaprenyl-phosphate phosphoribosyltransferase	5	4
	Multifunctional cyclase-dehydratase-3-O-methyl transferase TcmN	5	4
	putative transcriptional regulatory protein TcrX	5	4
	D-alanine--D-alanine ligase	5	4
	hypothetical protein	5	4
	hypothetical protein	5	4
	putative oxidoreductase	5	4
	hypothetical protein	5	4
**Melittangium**	Multifunctional cyclase-dehydratase-3-O-methyl transferase TcmN	4	3
	Acyl carrier protein	4	3
	Alpha-pyrone synthesis polyketide synthase-like Pks18	4	3
	Long-chain-fatty-acid--AMP ligase FadD29	4	3

	Alpha-pyrone synthesis polyketide synthase-like Pks11	4	3
	Proline--tRNA ligase	4	3
	High-affinity branched-chain amino acid transport ATP-binding protein LivF	4	3
	Lipopolysaccharide export system ATP-binding protein LptB	4	3
	hypothetical protein	4	3
	Methyl-accepting chemotaxis protein CtpH	4	3
**Myxococcus**	Circadian clock protein kinase KaiC	38	34
	Sporulation initiation phosphotransferase F	39	35
	putative lipoprotein YbbD precursor	39	35
	Putative peroxiredoxin bcp	39	35
	hypothetical protein	38	34
	hypothetical protein	39	36
	putative transcriptional regulatory protein TcrX	64	46
	hypothetical protein	38	35
	Multifunctional cyclase-dehydratase-3-O-methyl transferase TcmN	39	36
	Decaprenyl-phosphate phosphoribosyltransferase	39	36
	putative decaprenylphosphoryl-beta-D-ribose oxidase	39	36
	putative oxidoreductase	63	56
	hypothetical protein	39	35
	3-(3-hydroxy-phenyl)propionate/3-hydroxycinnamic acid hydroxylase	38	35
	Long-chain-fatty-acid--AMP ligase FadD29	38	35
	Meromycolate extension acyl carrier protein	39	38
	hypothetical protein	38	37
	Alpha-pyrone synthesis polyketide synthase-like Pks11	39	38
	Sulfate/thiosulfate import ATP-binding protein CysA	38	36
	Molybdenum transport system permease protein ModB	40	38
	Molybdate-binding periplasmic protein precursor	38	36
	Organic hydroperoxide resistance transcriptional regulator	38	36
	hypothetical protein	39	37
	hypothetical protein	37	36
	hypothetical protein	39	37

	hypothetical protein	38	36
	hypothetical protein	38	35
	hypothetical protein	37	34
	hypothetical protein	39	37
	Proline--tRNA ligase	62	40
	hypothetical protein	38	36
	hypothetical protein	38	36
	hypothetical protein	36	34
	hypothetical protein	36	33
	hypothetical protein	37	36
	hypothetical protein	37	34
	hypothetical protein	36	34
	hypothetical protein	36	33
**Pyxidicoccus**	hypothetical protein	7	5
	3-(3-hydroxy-phenyl)propionate/3-hydroxycinnamic acid hydroxylase	7	5
	Molybdenum transport system permease protein ModB	7	5
	Long-chain-fatty-acid--AMP ligase FadD29	7	5
	Glucose-1-phosphate adenylyltransferase	7	5
	hypothetical protein	7	5
	Sulfate/thiosulfate import ATP-binding protein CysA	5	5
	Alpha-pyrone synthesis polyketide synthase-like Pks11	5	5
	Multidrug resistance operon repressor	4	4
	hypothetical protein	3	3
	Molybdate-binding periplasmic protein precursor	3	3
	Transcriptional activator NphR	3	3
	Acyl carrier protein	3	3
**Stigmatella**	hypothetical protein	6	6
	putative oxidoreductase	6	6
	Alpha-pyrone synthesis polyketide synthase-like Pks11	6	6
	putative transcriptional regulatory protein TcrX	6	6

	Multifunctional cyclase-dehydratase-3-O-methyl transferase TcmN	6	6
	Proline--tRNA ligase	6	6
	Putative peroxiredoxin bcp	6	6
	Meromycolate extension acyl carrier protein	6	6
	hypothetical protein	6	6
	hypothetical protein	3	3
	Long-chain-fatty-acid--AMP ligase FadD26	3	3
	Decaprenyl-phosphate phosphoribosyltransferase	3	3
	putative decaprenylphosphoryl-beta-D-ribose oxidase	3	3
	Virulence sensor protein BvgS precursor	3	3
	3-(3-hydroxy-phenyl)propionate/3-hydroxycinnamic acid hydroxylase	3	3
	putative HTH-type transcriptional regulator YusO	3	3
	hypothetical protein	3	3
	hypothetical protein	3	3
	2'-5'-RNA ligase	3	3
	hypothetical protein	3	3
	hypothetical protein	3	3
	hypothetical protein	3	3
	hypothetical protein	3	3
	hypothetical protein	3	3
	putative HTH-type transcriptional regulator YusO	3	3
	Long-chain-fatty-acid--AMP ligase FadD26	3	3
	3-(3-hydroxy-phenyl)propionate/3-hydroxycinnamic acid hydroxylase	3	3
	putative decaprenylphosphoryl-beta-D-ribose oxidase	3	3
	Decaprenyl-phosphate phosphoribosyltransferase	3	3
	hypothetical protein	3	3
	hypothetical protein	3	3

Table S7. Unknown type I PKS BGC conserved features

type I PKS	conserved gene	# of strains	# in BGC

**Archangium**	Sporulation initiation phosphotransferase F	12	11
	Toluene 1,2-dioxygenase system ferredoxin subunit	12	11
	putative FAD-linked oxidoreductase	12	11
	RNA polymerase sigma factor SigA	12	11
	Acyl-CoA dehydrogenase	12	11
	hypothetical protein	11	10
	hypothetical protein	12	8
	hypothetical protein	10	8
	High-affinity zinc uptake system membrane protein ZnuB	10	9
	Erythronolide synthase, modules 1 and 2	9	8
	GTP cyclohydrolase 1	9	8
	Manganese ABC transporter substrate-binding lipoprotein precursor	9	8
	High-affinity zinc uptake system ATP-binding protein ZnuC	9	8
	Formyl-coenzyme A transferase	9	8
	hypothetical protein	8	7
	Long-chain-fatty-acid--CoA ligase FadD15	8	7
	Non-motile and phage-resistance protein	8	7
	hypothetical protein	7	6
	hypothetical protein	6	6
**Melittangium**	Erythronolide synthase, modules 1 and 2	3	3
	Phthiocerol synthesis polyketide synthase type I PpsC	3	3
	Acyl-CoA dehydrogenase	3	3
	hypothetical protein	3	3
	RNA polymerase sigma factor SigA	3	3
	putative FAD-linked oxidoreductase	3	3
	Zinc import ATP-binding protein ZnuC	3	3
	Manganese transport system membrane protein MntB	3	3

Table S8. Primer table

Primer Name	Sequence (5' to 3')	Product Size (bp)	Purpose

S_bpsA_F	ctcaaactagataccaggcatccgaaaggaagctgagttggctg	3971	Amplify *S. lavendulae bpsA* with overlap for pet-28a vector
S_bpsA_R	catcgtcgtttcctcgcatcgtggcatggtatatctccttcttaaagttaaac	3971	Amplify *S. lavendulae bpsA* with overlap for pet-28a vector
S_pet-28a_F	ctcaaactagataccaggcatccgaaaggaagctgagttggctg	5234	Amplify pet-28a vector with overlap for *S. lavendulae bpsA*
S_pet-28a_R	*catcgtcgtttcctcgcatcgtggcatggtatatctccttcttaaagttaaac*	5234	Amplify pet-28a vector with overlap for *S. lavendulae bpsA*
MbpsA_V1_F	gtttaactttaagaaggagatataccatgaatacggaaattctggcgaaagc	3966	Amplify *M. primigenium bpsA* with overlap for pet-28a vector
MbpsA_V1_R	cagccaactcagcttcctttcgggatgtcgtggattagcattggc	3966	Amplify *M. primigenium bpsA* with overlap for pet-28a vector
M_pet-28a_v1_F	gtttaactttaagaaggagatataccatgaatacggaaattctggcgaaagc	5234	Amplify pet-28a vector with overlap for *M. primigenium bpsA*
M_pet-28a_v1_R	gctttcgccagaatttccgtattcatggtatatctccttcttaaagttaaac	5234	Amplify pet-28a vector with overlap for *M. primigenium bpsA*
M_bpsA_v2_F	gccaatgctaatccacgacatcccgaaaggaagctgagttggctg	4105	Amplify *M. primigenium bpsA* with overlap for BAC vector
M_bpsA_v2_R	tggtatctagtttgagtctgcggatgtcgtggattagcattggc	4105	Amplify *M. primigenium bpsA* with overlap for BAC vector
S_*bpsA*_check_F	gaagagcaagctccaggtcaagg	958	Check for the presence of *bpsA* from *S. lavendulae*
S_*bpsA*_check_R	gttcatcatcaggtgctggagcttc	958	Check for the presence of *bpsA* from *S. lavendulae*
M_*bpsA*_check_F	gatcgagaaccacgactgggtc	1240	Check for the presence of *bpsA* from *M. primigenium*
M_*bpsA*_check_R	gaagaagtaggagggaccgctctc	1240	Check for the presence of *bpsA* from *M. primigenium*

Figure S1: Maximum likelihood tree generated from 16S rRNA gene sequence data from our isolates and all validly published type strain myxobacteria from the List of Prokaryotic names with Standing Nomenclature (LPSN) database. Tree rendered with MEGA 12.

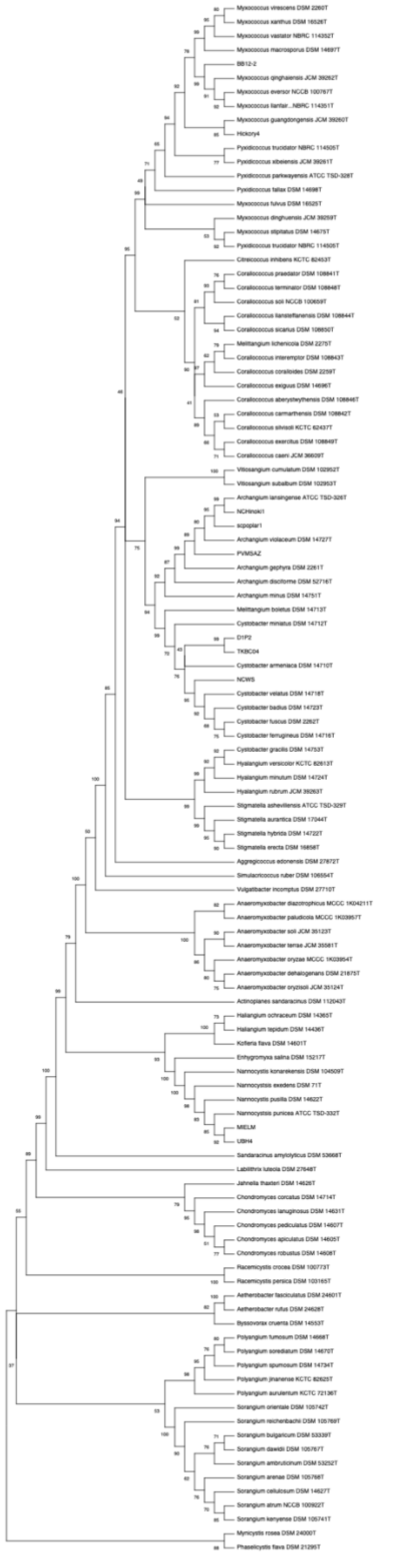

Table S9. Plasmid table

Plasmid name	Gene of interest	Vector	Antibiotic resistance	promotor	source
pNS001	*bpsA* sourced from *M. primigenium*	pet-28a	kanR	T7	This study
pNS002	*bpsA* sourced from *S. lavendulae*	pet-28a	kanR	T7	This study

Figure S2: Alignment of BpsA amino acid sequence data constructed with MEGA X using ClustalW.

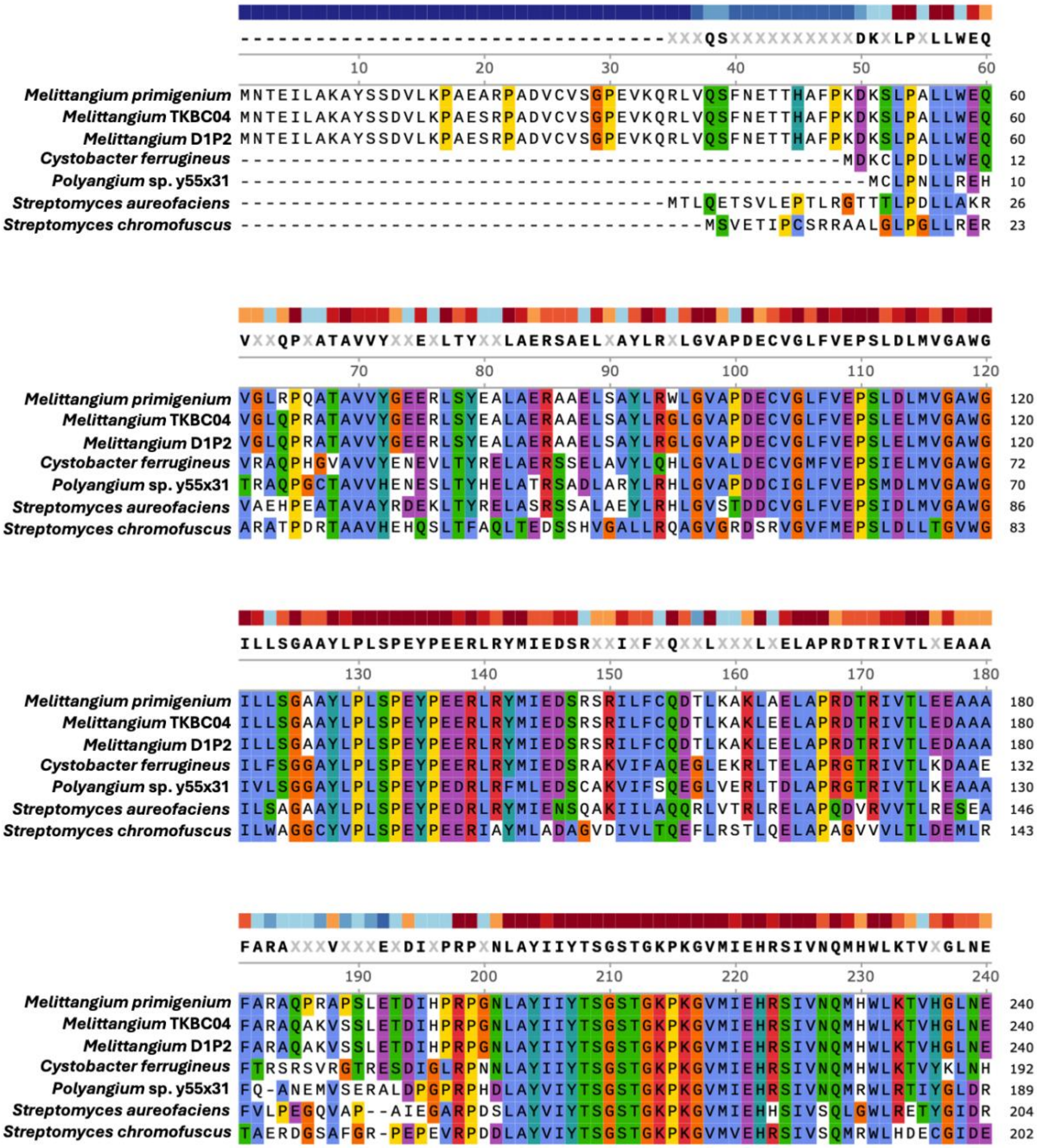

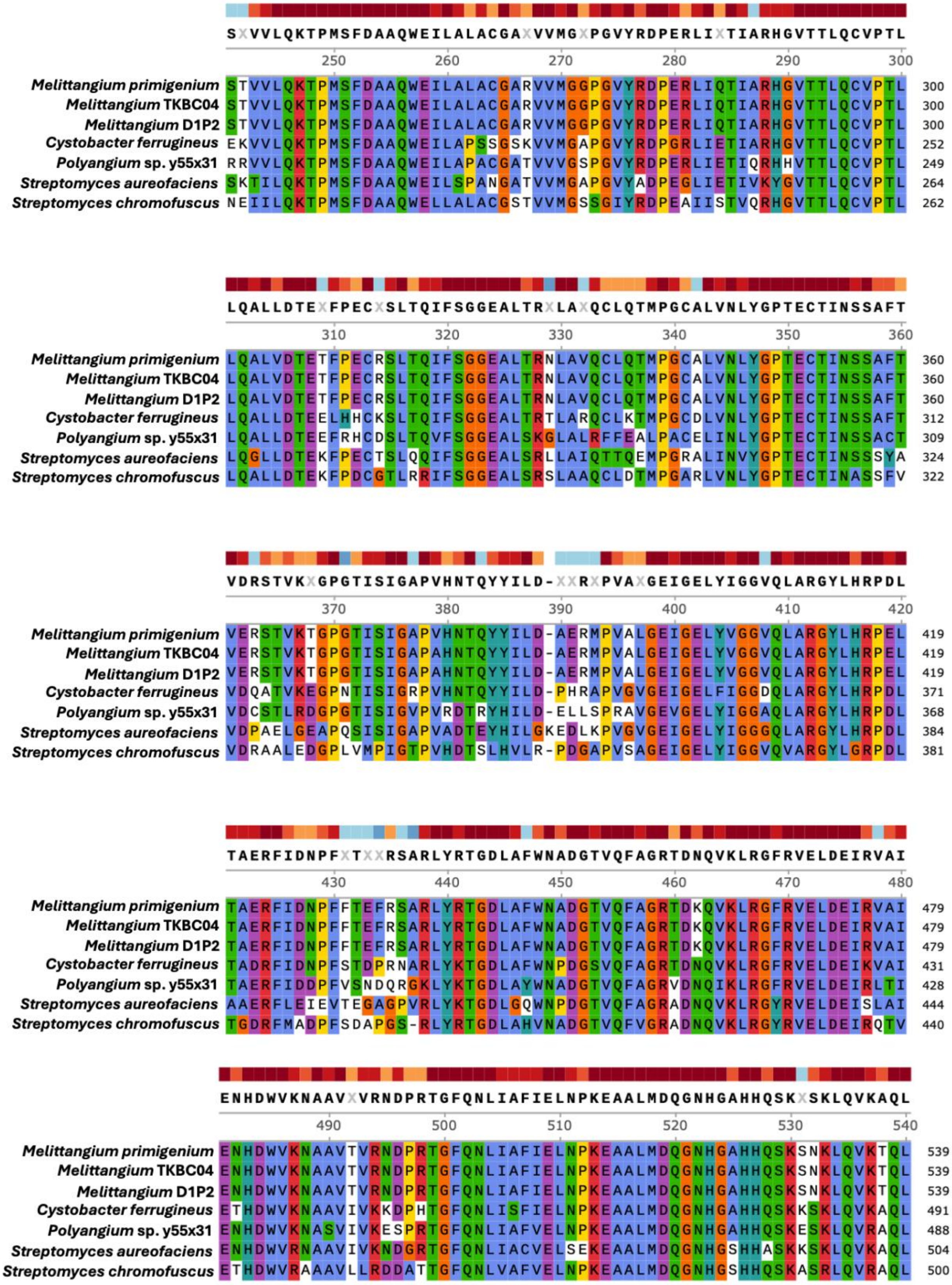
SXVVLQKTPMSFDAAQWEILALACGAXVVMGXPGVYRDPERLIXTIARHGVTTLQCVPTL
250 260 270 280 290 300
Melittangium primigenium STVVLQKTPMSFDAAQWEILALACGARVVMGGPGVYRDPERLIQTIARHGVTTLQCVPTL 300
Melittangium TKBC04 STVVLQKTPMSFDAAQWEILALACGARVVMGGPGVYRDPERLIQTIARHGVTTLQCVPTL 300
Melittangium D1P2 STVVLQKTPMSFDAAQWEILALACGARVVMGGPGVYRDPERLIQTIARHGVTTLQCVPTL 300
Cystobacter ferrugineus EKVVLQKTPMSFDAAQWEILAPSSGSKVVMGAPGVYRDPGRLIETIARHGVTTLQCVPTL 252
Polyangium sp. y55x31 RRVVLQKTPMSFDAAQWEILAPACGATVVVGSPGVYRDPERLIETIQRHHVTTLQCVPTL 249
Streptomyces aureofaciens SKTILQKTPMSFDAAQWEILSPANGATVVMGAPGVYADPEGLIETIVKYGVTTLQCVPTL 264
Streptomyces chromofuscus NEIILQKTPMSFDAAQWELLALACGSTVVMGSSGIYRDPEAIISTVQRHGVTTLQCVPTL 262
LQALLDTEXFPECXSLTQIFSGGEALTRXLAXQCLQTMPGCALVNLYGPTECTINSSAFT
310 320 330 340 350 360
Melittangium primigenium LQALVDTETFPECRSLTQIFSGGEALTRNLAVQCLQTMPGCALVNLYGPTECTINSSAFT 360
Melittangium TKBC04 LQALVDTETFPECRSLTQIFSGGEALTRNLAVQCLQTMPGCALVNLYGPTECTINSSAFT 360
Melittangium D1P2 LQALVDTETFPECRSLTQIFSGGEALTRNLAVQCLQTMPGCALVNLYGPTECTINSSAFT 360
Cystobacter ferrugineus LQALLDTEELHHCKSLTQIFSGGEALTRTLARQCLKTMPGCDLVNLYGPTECTINSSAFT 312
Polyangium sp. y55x31 LQALLDTEEFRHCDSLTQVFSGGEALSKGLALRFFEALPACELINLYGPTECTINSSACT 309
Streptomyces aureofaciens LQGLLDTEKFPECTSLQQIFSGGEALSRLLAIQTTQEMPGRALINVYGPTECTINSSSYA 324
Streptomyces chromofuscus LQALLDTEKFPDCGTLRRIFSGGEALSRSLAAQCLDTMPGARLVNLYGPTECTINASSFV 322
VDRSTVKXGPGTISIGAPVHNTQYYILD-XXRXPVAXGEIGELYIGGVQLARGYLHRPDL
370 380 390 400 410 420
Melittangium primigenium VERSTVKTGPGTISIGAPVHNTQYYILD-AERMPVALGEIGELYVGGVQLARGYLHRPEL 419
Melittangium TKBC04 VERSTVKTGPGTISIGAPAHNTQYYILD-AERMPVALGEIGELYVGGVQLARGYLHRPEL 419
Melittangium D1P2 VERSTVKTGPGTISIGAPAHNTQYYILD-AERMPVALGEIGELYVGGVQLARGYLHRPEL 419
Cystobacter ferrugineus VDQATVKEGPNTISIGRPVHNTQYYILD-PHRAPVGVGEIGELFIGGDQLARGYLHRPDL 371
Polyangium sp. y55x31 VDCSTLRDGPGTISIGVPVRDTRYHILD-ELLSPRAVGEVGELYIGGAQLARGYLHRPDL 368
Streptomyces aureofaciens VDPAELGEAPQSISIGAPVADTEYHILGKEDLKPVGVGEIGELYIGGGQLARGYLHRPDL 384
Streptomyces chromofuscus VDRAALEDGPLVMPIGTPVHDTSLHVLR-PDGAPVSAGEIGELYIGGVQVARGYLGRPDL 381
TAERFIDNPFXTXXRSARLYRTGDLAFWNADGTVQFAGRTDNQVKLRGFRVELDEIRVAI
430 440 450 460 470 480
Melittangium primigenium TAERFIDNPFFTEFRSARLYRTGDLAFWNADGTVQFAGRTDKQVKLRGFRVELDEIRVAI 479
Melittangium TKBC04 TAERFIDNPFFTEFRSARLYRTGDLAFWNADGTVQFAGRTDKQVKLRGFRVELDEIRVAI 479
Melittangium D1P2 TAERFIDNPFFTEFRSARLYRTGDLAFWNADGTVQFAGRTDKQVKLRGFRVELDEIRVAI 479
Cystobacter ferrugineus TADRFIDNPFSTDPRNARLYKTGDLAFWNPDGSVQFAGRTDNQVKLRGFRVELDEIKVAI 431
Polyangium sp. y55x31 TAERFIDDPFVSNDQRGKLYKTGDLAYWNADGTVQFAGRVDNQIKLRGFRVELDEIRLTI 428
Streptomyces aureofaciens AAERFLEIEVTEGAGPVRLYKTGDLGQWNPDGTVQFAGRADNQVKLRGYRVELDEISLAI 444
Streptomyces chromofuscus TGDRFMADPFSDAPGS-RLYRTGDLAHVNADGTVQFVGRADNQVKLRGYRVELDEIRQTV 440
ENHDWVKNAAVXVRNDPRTGFQNLIAFIELNPKEAALMDQGNHGAHHQSKXSKLQVKAQL
490 500 510 520 530 540
Melittangium primigenium ENHDWVKNAAVTVRNDPRTGFQNLIAFIELNPKEAALMDQGNHGAHHQSKSNKLQVKTQL 539
Melittangium TKBC04 ENHDWVKNAAVTVRNDPRTGFQNLIAFIELNPKEAALMDQGNHGAHHQSKSNKLQVKTQL 539
Melittangium D1P2 ENHDWVKNAAVTVRNDPRTGFQNLIAFIELNPKEAALMDQGNHGAHHQSKSNKLQVKTQL 539
Cystobacter ferrugineus ETHDWVKNAAVIVKKDPHTGFQNLISFIELNPKEAALMDQGNHGAHHQSKKSKLQVKAQL 491
Polyangium sp. y55x31 ENHDWVKNASVIVKESPRTGFQNLIAFVELNPKEAALMDQGNHGAHHQSKESKLQVRAQL 488
Streptomyces aureofaciens ENHDWVRNAAVIVKNDGRTGFQNLIACVELSEKEAALMDQGNHGSHHASKKSKLQVKAQL 504
Streptomyces chromofuscus ETHDWVRAAAVLLRDDATTGFQNLVAFVELNPKEAALMDQGNHGSHHQSKASRLQVRAQL 500

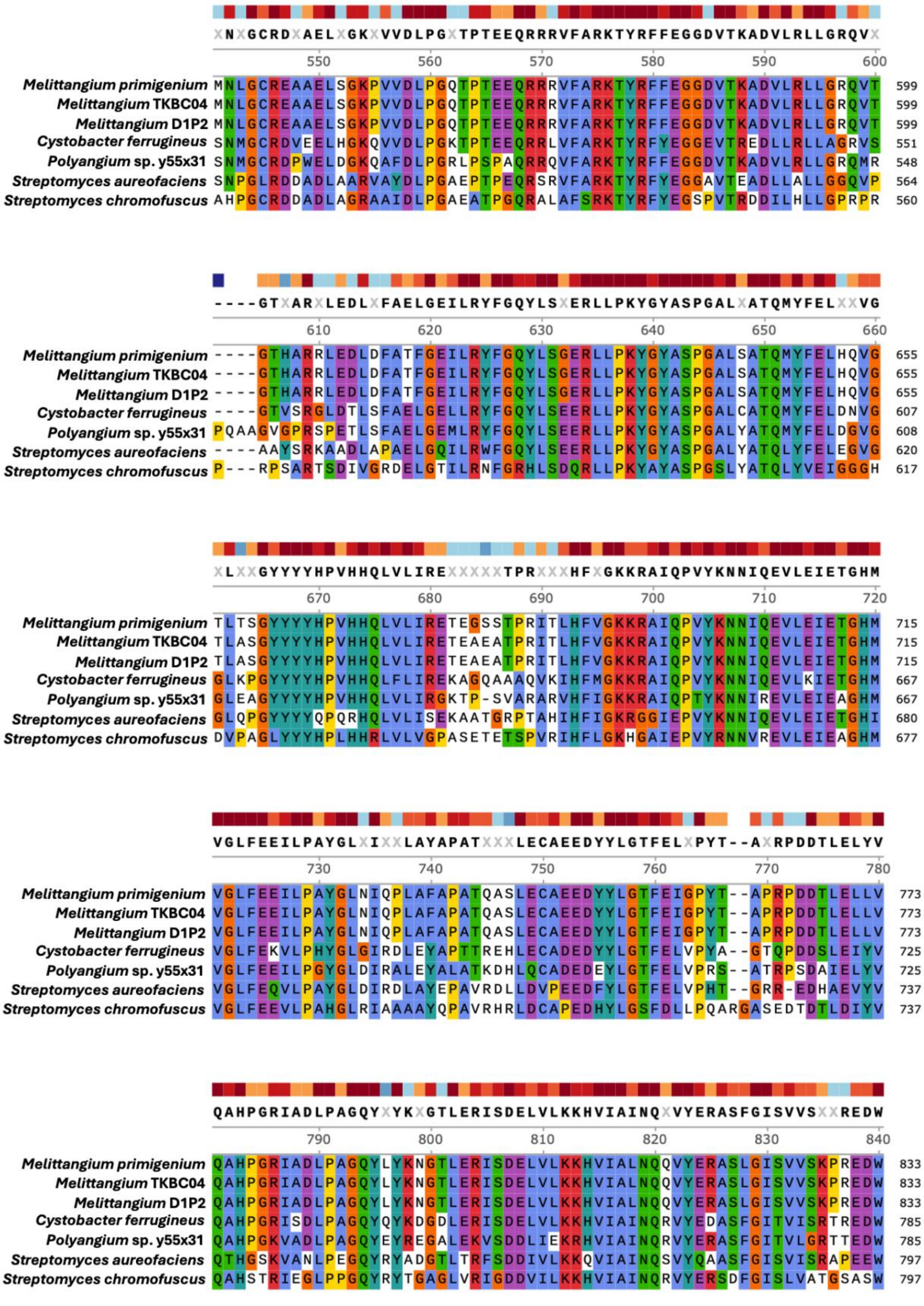
XNXGCRDXAELXGKXVVDLPGXTPTEEQRRRVFARKTYRFFEGGDVTKADVLRLLGRQVX
550 560 570 580 590 600
Melittangium primigenium MNLGCREAAELSGKPVVDLPGQTPTEEQRRRVFARKTYRFFEGGDVTKADVLRLLGRQVT 599
Melittangium TKBC04 MNLGCREAAELSGKPVVDLPGQTPTEEQRRRVFARKTYRFFEGGDVTKADVLRLLGRQVT 599
Melittangium D1P2 MNLGCREAAELSGKPVVDLPGQTPTEEQRRRVFARKTYRFFEGGDVTKADVLRLLGRQVT 599
Cystobacter ferrugineus SNMGCRDVEELHGKQVVDLPGKTPTEEQRRLVFARKTYRFYEGGEVTREDLLRLLAGRVS 551
Polyangium sp. y55x31 SNMGCRDPWELDGKQAFDLPGRLPSPAQRRQVFARKTYRFFEGGDVTKADVLRLLGRQMR 548
Streptomyces aureofaciens SNPGLRDDADLAARVAYDLPGAEPTPEQRSRVFARKTYRFYEGGAVTEADLLALLGGQVP 564
Streptomyces chromofuscus AHPGCRDDADLAGRAAIDLPGAEATPGQRALAFSRKTYRFYEGSPVTRDDILHLLGPRPR 560
----GTXARXLEDLXFAELGEILRYFGQYLSXERLLPKYGYASPGALXATQMYFELXXVG
610 620 630 640 650 660
Melittangium primigenium ----GTHARRLEDLDFATFGEILRYFGQYLSGERLLPKYGYASPGALSATQMYFELHQVG 655
Melittangium TKBC04 ----GTHARRLEDLDFATFGEILRYFGQYLSGERLLPKYGYASPGALSATQMYFELHQVG 655
Melittangium D1P2 ----GTHARRLEDLDFATFGEILRYFGQYLSGERLLPKYGYASPGALSATQMYFELHQVG 655
Cystobacter ferrugineus ----GTVSRGLDTLSFAELGELLRYFGQYLSEERLLPKYGYASPGALCATQMYFELDNVG 607
Polyangium sp. y55x31 PQAAGVGPRSPETLSFAELGEMLRYFGQYLSEERLLPKYGYASPGALYATQMYFELDGVG 608
Streptomyces aureofaciens ----AAYSRKAADLAPAELGQILRWFGQYLSEERLLPKYGYASPGALYATQLYFELEGVG 620
Streptomyces chromofuscus P---RPSARTSDIVGRDELGTILRNFGRHLSDQRLLPKYAYASPGSLYATQLYVEIGGGH 617
XLXXGYYYYHPVHHQLVLIREXXXXXTPRXXXHFXGKKRAIQPVYKNNIQEVLEIETGHM
670 680 690 700 710 720
Melittangium primigenium TLTSGYYYYHPVHHQLVLIRETEGSSTPRITLHFVGKKRAIQPVYKNNIQEVLEIETGHM 715
Melittangium TKBC04 TLASGYYYYHPVHHQLVLIRETEAEATPRITLHFVGKKRAIQPVYKNNIQEVLEIETGHM 715
Melittangium D1P2 TLASGYYYYHPVHHQLVLIRETEAEATPRITLHFVGKKRAIQPVYKNNIQEVLEIETGHM 715
Cystobacter ferrugineus GLKPGYYYYHPVHHQLFLIREKAGQAAAQVKIHFMGKKRAIQPVYKNNIQEVLKIETGHM 667
Polyangium sp. y55x31 GLEAGYYYYHPVHHQLVLIRGKTP-SVARARVHFIGKKRAIQPTYKNNIREVLEIEAGHM 667
Streptomyces aureofaciens GLQPGYYYYQPQRHQLVLISEKAATGRPTAHIHFIGKRGGIEPVYKNNIQEVLEIETGHI 680
Streptomyces chromofuscus DVPAGLYYYHPLHHRLVLVGPASETETSPVRIHFLGKHGAIEPVYRNNVREVLEIEAGHM 677
VGLFEEILPAYGLXIXXLAYAPATXXXLECAEEDYYLGTFELXPYT--AXRPDDTLELYV
730 740 750 760 770 780
Melittangium primigenium VGLFEEILPAYGLNIQPLAFAPATQASLECAEEDYYLGTFEIGPYT--APRPDDTLELLV 773
Melittangium TKBC04 VGLFEEILPAYGLNIQPLAFAPATQASLECAEEDYYLGTFEIGPYT--APRPDDTLELLV 773
Melittangium D1P2 VGLFEEILPAYGLNIQPLAFAPATQASLECAEEDYYLGTFEIGPYT--APRPDDTLELLV 773
Cystobacter ferrugineus VGLFEKVLPHYGLGIRDLEYAPTTREHLECADEDYYLGTFELVPYA--GTQPDDSLEIYV 725
Polyangium sp. y55x31 VGLFEEILPGYGLDIRALEYALATKDHLQCADEDEYLGTFELVPRS--ATRPSDAIELYV 725
Streptomyces aureofaciens VGLFEQVLPAYGLDIRDLAYEPAVRDLLDVPEEDFYLGTFELVPHT--GRR-EDHAEVYV 737
Streptomyces chromofuscus VGLFEEVLPAHGLRIAAAAYQPAVRHRLDCAPEDHYLGSFDLLPQARGASEDTDTLDIYV 737
QAHPGRIADLPAGQYXYKXGTLERISDELVLKKHVIAINQXVYERASFGISVVSXXREDW
790 800 810 820 830 840
Melittangium primigenium QAHPGRIADLPAGQYLYKNGTLERISDELVLKKHVIALNQQVYERASLGISVVSKPREDW 833
Melittangium TKBC04 QAHPGRIADLPAGQYLYKNGTLERISDELVLKKHVIALNQQVYERASLGISVVSKPREDW 833
Melittangium D1P2 QAHPGRIADLPAGQYLYKNGTLERISDELVLKKHVIALNQQVYERASLGISVVSKPREDW 833
Cystobacter ferrugineus QAHPGRISDLPAGQYQYKDGDLERISDELVLKKHVIAINQRVYEDASFGITVISRTREDW 785
Polyangium sp. y55x31 QAHPGKVADLPAGQYEYREGALEKVSDDLIEKRHVIAINQRVYERASFGITVLGRTTEDW 785
Streptomyces aureofaciens QTHGSKVANLPEGQYRYADGTLTRFSDDIVLKKQVIAINQSVYQAASFGISVISRAPEEW 797
Streptomyces chromofuscus QAHSTRIEGLPPGQYRYTGAGLVRIGDDVILKKHVIAINQRVYERSDFGISLVATGSASW 797

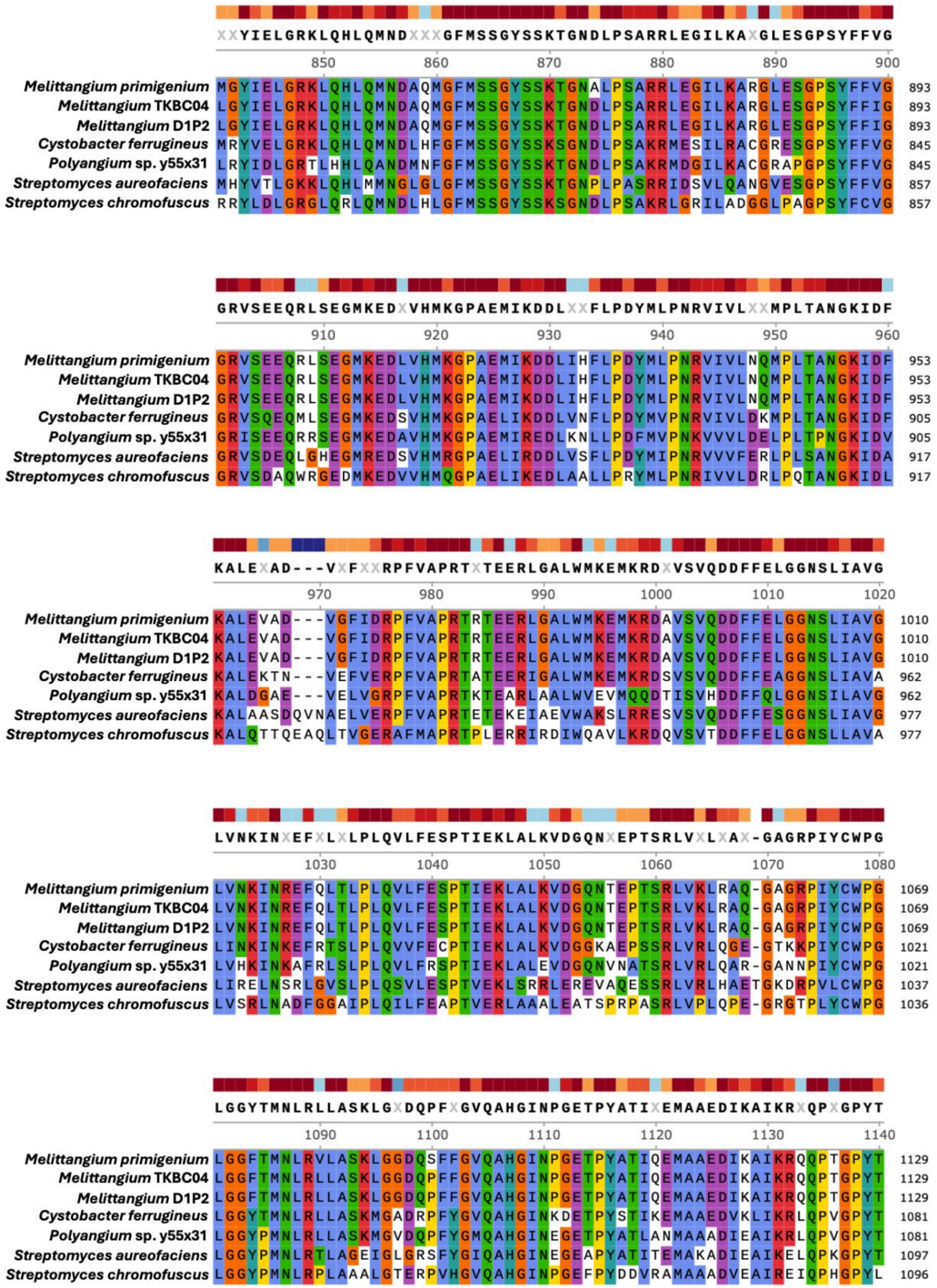
XXYIELGRKLQHLQMNDXXXGFMSSGYSSKTGNDLPSARRLEGILKAXGLESGPSYFFVG
850 860 870 880 890 900
Melittangium primigenium MGYIELGRKLQHLQMNDAQMGFMSSGYSSKTGNALPSARRLEGILKARGLESGPSYFFVG 893
Melittangium TKBC04 LGYIELGRKLQHLQMNDAQMGFMSSGYSSKTGNDLPSARRLEGILKARGLESGPSYFFIG 893
Melittangium D1P2 LGYIELGRKLQHLQMNDAQMGFMSSGYSSKTGNDLPSARRLEGILKARGLESGPSYFFIG 893
Cystobacter ferrugineus MRYVELGRKLQHLQMNDLHFGFMSSGYSSKSGNDLPSAKRMESILRACGRESGPSYFFVG 845
Polyangium sp. y55x31 LRYIDLGRTLHHLQANDMNFGFMSSGYSSKTGNDLPSAKRMDGILKACGRAPGPSYFFVG 845
Streptomyces aureofaciens MHYVTLGKKLQHLMMNGLGLGFMSSGYSSKTGNPLPASRRIDSVLQANGVESGPSYFFVG 857
Streptomyces chromofuscus RRYLDLGRGLQRLQMNDLHLGFMSSGYSSKSGNDLPSAKRLGRILADGGLPAGPSYFCVG 857
GRVSEEQRLSEGMKEDXVHMKGPAEMIKDDLXXFLPDYMLPNRVIVLXXMPLTANGKIDF
910 920 930 940 950 960
Melittangium primigenium GRVSEEQRLSEGMKEDLVHMKGPAEMIKDDLIHFLPDYMLPNRVIVLNQMPLTANGKIDF 953
Melittangium TKBC04 GRVSEEQRLSEGMKEDLVHMKGPAEMIKDDLIHFLPDYMLPNRVIVLNQMPLTANGKIDF 953
Melittangium D1P2 GRVSEEQRLSEGMKEDLVHMKGPAEMIKDDLIHFLPDYMLPNRVIVLNQMPLTANGKIDF 953
Cystobacter ferrugineus GRVSQEQMLSEGMKEDSVHMKGPAELIKDDLVNFLPDYMVPNRVIVLDKMPLTANGKIDF 905
Polyangium sp. y55x31 GRISEEQRRSEGMKEDAVHMKGPAEMIREDLKNLLPDFMVPNKVVVLDELPLTPNGKIDV 905
Streptomyces aureofaciens GRVSDEQLGHEGMREDSVHMRGPAELIRDDLVSFLPDYMIPNRVVVFERLPLSANGKIDA 917
Streptomyces chromofuscus GRVSDAQWRGEDMKEDVVHMQGPAELIKEDLAALLPRYMLPNRIVVLDRLPQTANGKIDL 917
KALEXAD---VXFXXRPFVAPRTXTEERLGALWMKEMKRDXVSVQDDFFELGGNSLIAVG
970 980 990 1000 1010 1020
Melittangium primigenium KALEVAD---VGFIDRPFVAPRTRTEERLGALWMKEMKRDAVSVQDDFFELGGNSLIAVG 1010
Melittangium TKBC04 KALEVAD---VGFIDRPFVAPRTRTEERLGALWMKEMKRDAVSVQDDFFELGGNSLIAVG 1010
Melittangium D1P2 KALEVAD---VGFIDRPFVAPRTRTEERLGALWMKEMKRDAVSVQDDFFELGGNSLIAVG 1010
Cystobacter ferrugineus KALEKTN---VEFVERPFVAPRTATEERIGALWMKEMKRDSVSVQDDFFEAGGNSLIAVA 962
Polyangium sp. y55x31 KALDGAE---VELVGRPFVAPRTKTEARLAALWVEVMQQDTISVHDDFFQLGGNSILAVG 962
Streptomyces aureofaciens KALAASDQVNAELVERPFVAPRTETEKEIAEVWAKSLRRESVSVQDDFFESGGNSLIAVG 977
Streptomyces chromofuscus KALQTTQEAQLTVGERAFMAPRTPLERRIRDIWQAVLKRDQVSVTDDFFELGGNSLLAVA 977
LVNKINXEFXLXLPLQVLFESPTIEKLALKVDGQNXEPTSRLVXLXAX-GAGRPIYCWPG
1030 1040 1050 1060 1070 1080
Melittangium primigenium LVNKINREFQLTLPLQVLFESPTIEKLALKVDGQNTEPTSRLVKLRAQ-GAGRPIYCWPG 1069
Melittangium TKBC04 LVNKINREFQLTLPLQVLFESPTIEKLALKVDGQNTEPTSRLVKLRAQ-GAGRPIYCWPG 1069
Melittangium D1P2 LVNKINREFQLTLPLQVLFESPTIEKLALKVDGQNTEPTSRLVKLRAQ-GAGRPIYCWPG 1069
Cystobacter ferrugineus LINKINKEFRTSLPLQVVFECPTIEKLALKVDGGKAEPSSRLVRLQGE-GTKKPIYCWPG 1021
Polyangium sp. y55x31 LVHKINKAFRLSLPLQVLFRSPTIEKLALEVDGQNVNATSRLVRLQAR-GANNPIYCWPG 1021
Streptomyces aureofaciens LIRELNSRLGVSLPLQSVLESPTVEKLSRRLEREVAQESSRLVRLHAETGKDRPVLCWPG 1037
Streptomyces chromofuscus LVSRLNADFGGAIPLQILFEAPTVERLAAALEATSPRPASRLVPLQPE-GRGTPLYCWPG 1036
LGGYTMNLRLLASKLGXDQPFXGVQAHGINPGETPYATIXEMAAEDIKAIKRXQPXGPYT
1090 1100 1110 1120 1130 1140
Melittangium primigenium LGGFTMNLRVLASKLGGDQSFFGVQAHGINPGETPYATIQEMAAEDIKAIKRQQPTGPYT 1129
Melittangium TKBC04 LGGFTMNLRLLASKLGGDQPFFGVQAHGINPGETPYATIQEMAAEDIKAIKRQQPTGPYT 1129
Melittangium D1P2 LGGFTMNLRLLASKLGGDQPFFGVQAHGINPGETPYATIQEMAAEDIKAIKRQQPTGPYT 1129
Cystobacter ferrugineus LGGYTMNLRLLASKMGADRPFYGVQAHGINKDETPYSTIKEMAAEDVKLIKRLQPVGPYT 1081
Polyangium sp. y55x31 LGGYPMNLRLLASKMGVDQPFYGMQAHGINEGETPYATLANMAAADIEAIKRLQPVGPYT 1081
Streptomyces aureofaciens LGGYPMNLRTLAGEIGLGRSFYGIQAHGINEGEAPYATITEMAKADIEAIKELQPKGPYT 1097
Streptomyces chromofuscus LGGYPMNLRPLAAALGTERPVHGVQAHGINPGEFPYDDVRAMAAADVEAIREIQPHGPYL 1096

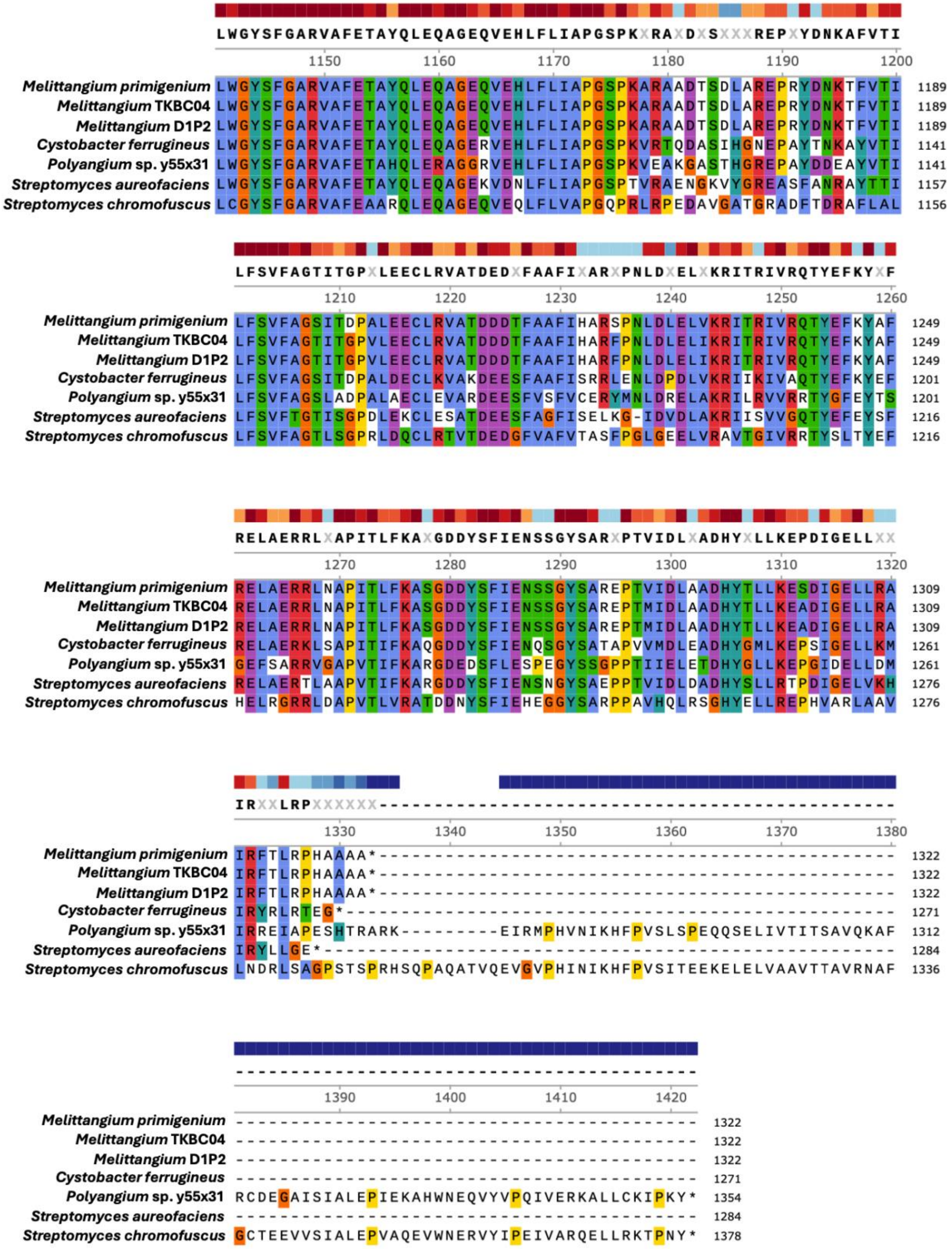

LWGYSFGARVAFETAYQLEQAGEQVEHLFLIAPGSPKXRAXDXSXXXREPXYDNKAFVTI
1150 1160 1170 1180 1190 1200
Melittangium primigenium LWGYSFGARVAFETAYQLEQAGEQVEHLFLIAPGSPKARAADTSDLAREPRYDNKTFVTI 1189
Melittangium TKBC04 LWGYSFGARVAFETAYQLEQAGEQVEHLFLIAPGSPKARAADTSDLAREPRYDNKTFVTI 1189
Melittangium D1P2 LWGYSFGARVAFETAYQLEQAGEQVEHLFLIAPGSPKARAADTSDLAREPRYDNKTFVTI 1189
Cystobacter ferrugineus LWGYSFGARVAFETAYQLEQAGERVEHLFLIAPGSPKVRTQDASIHGNEPAYTNKAYVTI 1141
Polyangium sp. y55x31 LWGYSFGARVAFETAHQLERAGGRVEHLFLIAPGSPKVEAKGASTHGREPAYDDEAYVTI 1141
Streptomyces aureofaciens LWGYSFGARVAFETAYQLEQAGEKVDNLFLIAPGSPTVRAENGKVYGREASFANRAYTTI 1157
Streptomyces chromofuscus LCGYSFGARVAFEAARQLEQAGEQVEQLFLVAPGQPRLRPEDAVGATGRADFTDRAFLAL 1156
LFSVFAGTITGPXLEECLRVATDEDXFAAFIXARXPNLDXELXKRITRIVRQTYEFKYXF
1210 1220 1230 1240 1250 1260
Melittangium primigenium LFSVFAGSITDPALEECLRVATDDDTFAAFIHARSPNLDLELVKRITRIVRQTYEFKYAF 1249
Melittangium TKBC04 LFSVFAGTITGPVLEECLRVATDDDTFAAFIHARFPNLDLELIKRITRIVRQTYEFKYAF 1249
Melittangium D1P2 LFSVFAGTITGPVLEECLRVATDDDTFAAFIHARFPNLDLELIKRITRIVRQTYEFKYAF 1249
Cystobacter ferrugineus LFSVFAGSITDPALDECLKVAKDEESFAAFISRRLENLDPDLVKRIIKIVAQTYEFKYEF 1201
Polyangium sp. y55x31 LFSVFAGSLADPALAECLEVARDEESFVSFVCERYMNLDRELAKRILRVVRRTYGFEYTS 1201
Streptomyces aureofaciens LFSVFTGTISGPDLEKCLESATDEESFAGFISELKG-IDVDLAKRIISVVGQTYEFEYSF 1216
Streptomyces chromofuscus LFSVFAGTLSGPRLDQCLRTVTDEDGFVAFVTASFPGLGEELVRAVTGIVRRTYSLTYEF 1216
RELAERRLXAPITLFKAXGDDYSFIENSSGYSARXPTVIDLXADHYXLLKEPDIGELLXX
1270 1280 1290 1300 1310 1320
Melittangium primigenium RELAERRLNAPITLFKASGDDYSFIENSSGYSAREPTVIDLAADHYTLLKESDIGELLRA 1309
Melittangium TKBC04 RELAERRLNAPITLFKASGDDYSFIENSSGYSAREPTMIDLAADHYTLLKEADIGELLRA 1309
Melittangium D1P2 RELAERRLNAPITLFKASGDDYSFIENSSGYSAREPTMIDLAADHYTLLKEADIGELLRA 1309
Cystobacter ferrugineus RELAERKLSAPITIFKAQGDDYSFIENQSGYSATAPVVMDLEADHYGMLKEPSIGELLKM 1261
Polyangium sp. y55x31 GEFSARRVGAPVTIFKARGDEDSFLESPEGYSSGPPTIIELETDHYGLLKEPGIDELLDM 1261
Streptomyces aureofaciens RELAERTLAAPVTIFKARGDDYSFIENSNGYSAEPPTVIDLDADHYSLLRTPDIGELVKH 1276
Streptomyces chromofuscus HELRGRRLDAPVTLVRATDDNYSFIEHEGGYSARPPAVHQLRSGHYELLREPHVARLAAV 1276
IRXXLRPXXXXXX---
1330 1340 1350 1360 1370 1380
Melittangium primigenium IRFTLRPHAAAAA*-- 1322
Melittangium TKBC04 IRFTLRPHAAAAA*-- 1322
Melittangium D1P2 IRFTLRPHAAAAA*-- 1322
Cystobacter ferrugineus IRYRLRTEG*-- 1271
Polyangium sp. y55x31 IRREIAPESHTRARK---------EIRMPHVNIKHFPVSLSPEQQSELIVTITSAVQKAF 1312
Streptomyces aureofaciens IRYLLGE*-- 1284
Streptomyces chromofuscus LNDRLSAGPSTSPRHSQPAQATVQEVGVPHINIKHFPVSITEEKELELVAAVTTAVRNAF 1336

1390 1400 1410 1420
Melittangium primigenium -- 1322
Melittangium TKBC04 -- 1322
Melittangium D1P2 -- 1322
Cystobacter ferrugineus -- 1271
Polyangium sp. y55x31 RCDEGAISIALEPIEKAHWNEQVYVPQIVERKALLCKIPKY* 1354
Streptomyces aureofaciens -- 1284
Streptomyces chromofuscus GCTEEVVSIALEPVAQEVWNERVYIPEIVARQELLRKTPNY* 1378

Figure S3: Alienhunter (v.1.7) analysis of the indigoidine BGC from *Me. primigenium* with predicted horizontal gene transfer events and BLAST+ (v.2.16.0) analysis for comparison with the *Streptomyces aureofaciens* and *Streptomyces chromofuscus* indigoidine BGCs (1). Image rendered with Proksee (2).

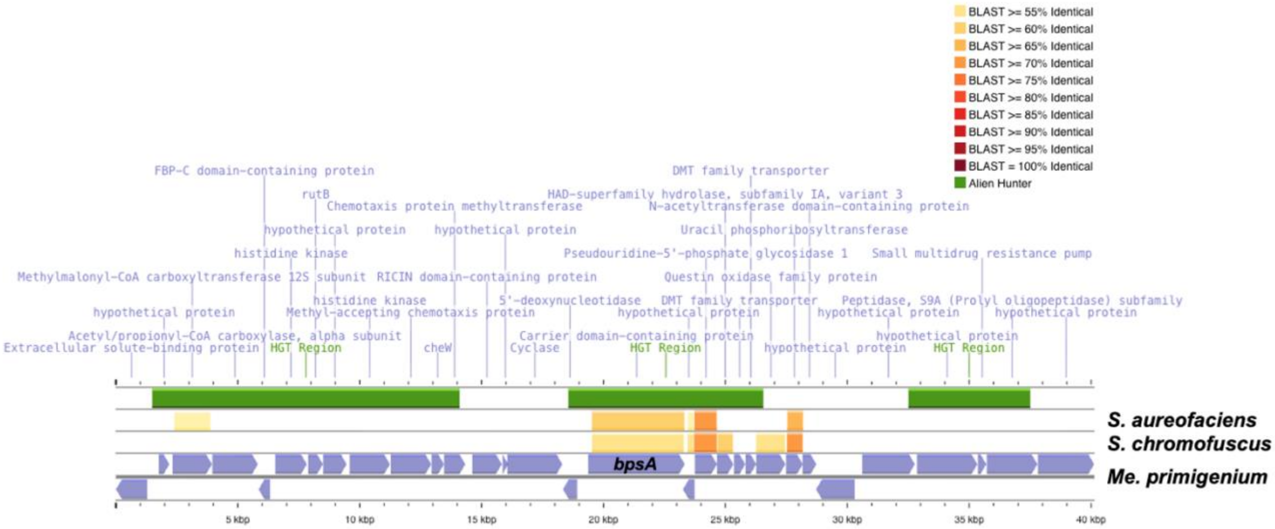

Figure S4: MS/MS spectra showing fragmentation data from (A) an indigoidine standard and (B) indigoidine produced by heterologous expression of BpsA from *Me. primigenium.*

A

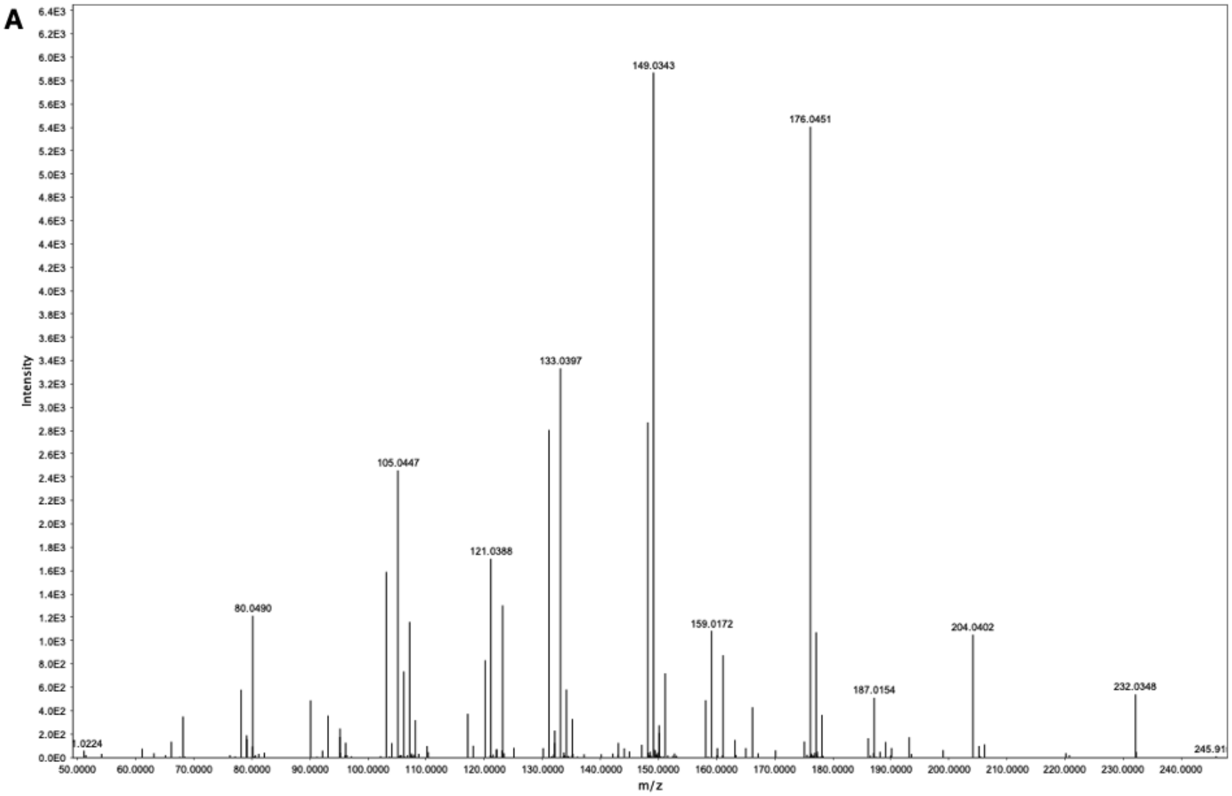

B

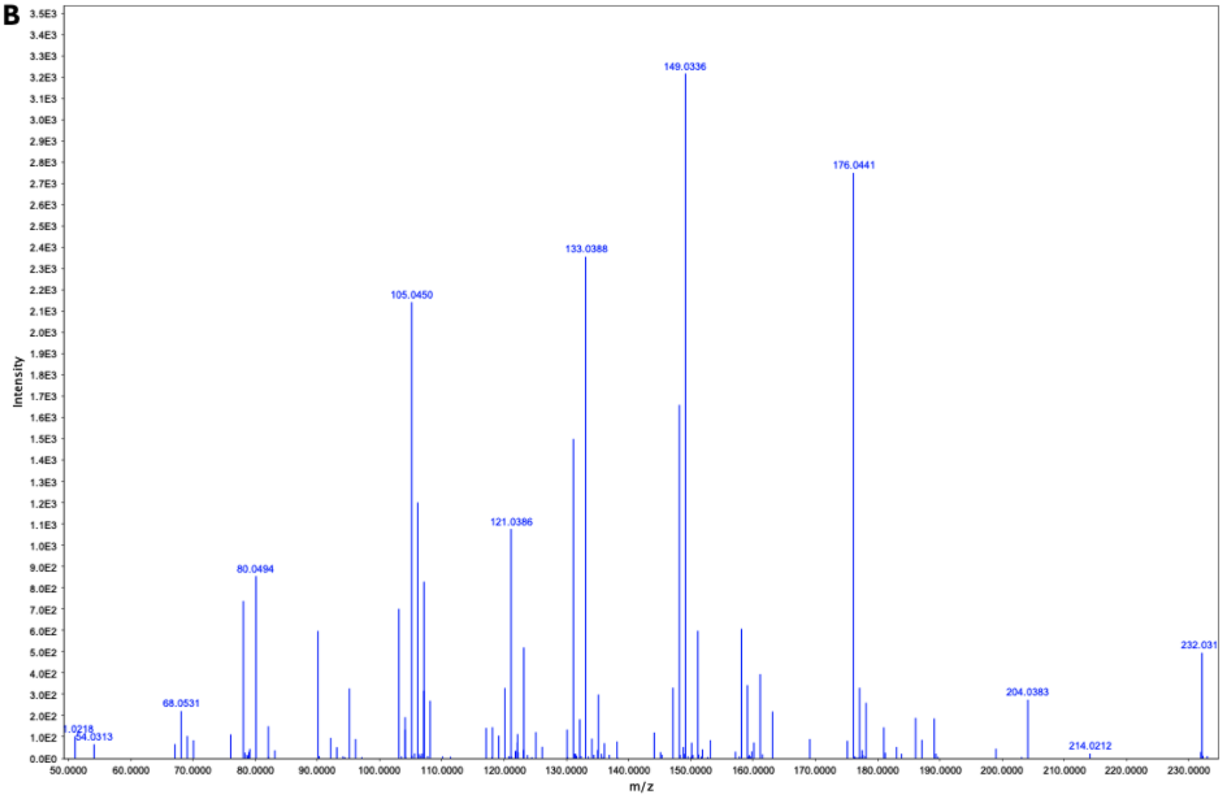

References

1. Vernikos GS, Parkhill J. 2006. Interpolated variable order motifs for identification of horizontally acquired DNA: revisiting the Salmonella pathogenicity islands. Bioinformatics. 22(18):2196-2203. doi: 10.1093/bioinformatics/btl369
2. Grant JR, Marinier E, Mandal A, Herman EK, Chen C, Graham M, Van Domselaar G, Stothard P. 2023. Proksee: in-depth characterization and visualization of bacterial genomes. Nucleic Acids Research gkad326, https://doi.org/10.1093/nar/gkad326
